# Interneuron migration impairment and brain region-specific DNA damage response following irradiation during early neurogenesis in mice

**DOI:** 10.1007/s00018-025-05643-7

**Published:** 2025-03-17

**Authors:** Lisa Berden, Nicholas Rajan, André Claude Mbouombouo Mfossa, Isabeau De Bie, Emre Etlioglu, Mohammed Abderrafi Benotmane, Mieke Verslegers, Najat Aourz, Ilse Smolders, Jean-Michel Rigo, Bert Brône, Roel Quintens

**Affiliations:** 1 Radiobiology Unit, Nuclear Medical Applications Institute, Belgian Nuclear Research Centre (SCK CEN), Mol, Belgium; 2 Laboratory for Neurophysiology, BIOMED Research Institute, UHasselt, Hasselt, Belgium; 3 4BRAIN, Department of Head and Skin, Faculty of Medicine and Health Sciences, Ghent University, Ghent, Belgium; 4 Preclinical Sciences and Translational Safety, Johnson & Johnson IM, Beerse, Belgium; 5 Research Group Experimental Pharmacology (EFAR), Center for Neurosciences (C4N), Faculteit Geneeskunde en Farmacie, Vrije Universiteit Brussel (VUB), Brussels, Belgium

**Keywords:** DNA damage, Neurodevelopment, Microcephaly, Interneuron migration, Seizures

## Abstract

Embryonic DNA damage resulting from DNA repair deficiencies or exposure to ionizing radiation during early neurogenesis can lead to neurodevelopmental disorders, including microcephaly. This has been linked to an excessive DNA damage response in dorsal neural progenitor cells (NPCs), resulting in p53-dependent apoptosis and premature neuronal differentiation which culminates in depletion of the NPC pool. However, the effect of DNA damage on ventral forebrain NPCs, the origin of interneurons, remains unclear. In this study, we investigated the sequelae of irradiation of mouse fetuses at an early timepoint of forebrain neurogenesis. We focused on the neocortex (NCX) and medial ganglionic eminence (MGE), key regions for developing dorsal and ventral NPCs, respectively. Although both regions showed a typical p53-mediated DNA damage response consisting of cell cycle arrest, DNA repair and apoptosis, NCX cells displayed prolonged cell cycle arrest, while MGE cells exhibited more sustained apoptosis. Moreover, irradiation reduced the migration speed of interneurons in acute living brain slices and MGE explants, the latter indicating a cell-intrinsic component in the defect. RNA sequencing and protein analyses revealed disruptions in actin and microtubule cytoskeletal-related cellular machinery, particularly in MGE cells. Despite massive acute apoptosis and an obvious interneuron migration defect, prenatally irradiated animals did not show increased sensitivity to pentylenetetrazole-induced seizures, nor was there a reduction in cortical interneurons in young adult mice. This suggests a high plasticity of the developing brain to acute insults during early neurogenesis. Overall, our findings indicate that embryonic DNA damage induces region-specific responses, potentially linked to neurodevelopmental disorders.

**Supplementary Information:**

The online version contains supplementary material available at 10.1007/s00018-025-05643-7.

## Introduction

Early brain development involves critically coordinated developmental processes, including neurogenesis, which requires high rates of cell proliferation, making the developing embryonic brain particularly sensitive to DNA damage [1, 2]. DNA repair deficiencies, mutations in microcephaly primary hereditary (MCPH) genes and prenatal exposure to ethanol, Zika virus (ZIKV), hypoxia and irradiation have been shown to generate highly reactive free radicals that induce reactive oxygen species, DNA damage and/or oxidative stress, leading to neurodevelopmental disorders such as microcephaly and/or (epileptic) seizures [3–11]. The cellular DNA damage response typically involves cell cycle arrest to allow for DNA repair through different pathways depending on the type and extent of the damage, and the cell cycle stage [12, 13]. If the damage is irreparable, cells may undergo apoptosis, senescence or differentiation as ways to escape malignancy. One of the central regulators of the DNA damage response is p53, a transcriptional activator of genes involved in cell cycle arrest, DNA repair and apoptosis [14].

Previously, it has been shown that dorsal and ventral oligodendrocyte progenitor cells (OPCs) exhibit distinct DNA damage responses following DNA damage [15]. Similarly, a differential response has been observed between early neuroepithelial cells and fate-committed neural progenitors under genotoxic stress [16]. Neural progenitor cells (NPCs) display a higher incidence of double-strand breaks (DSBs) compared to neurons when exposed to topoisomerase II inhibitors, and are also more radiosensitive than neurons [17, 18]. These findings suggest that the sensitivity to DNA damage and the subsequent DNA damage response varies depending on the cell type, their cell cycle properties, developmental stage and location. However, while microcephaly in mice irradiated during the earlier stages of neurogenesis (embryonic day (E) 11 to 14.5) has been attributed to apoptosis and/or premature neuronal differentiation of dorsal NPCs [17, 19–22], the contribution of ventral forebrain NPCs has not yet been explored.

During early neurogenesis in mice, dorsal forebrain excitatory neurons are predominantly derived from intermediate progenitors, or by asymmetric divisions of NPCs between E11-E13. These neurons then migrate radially in an inside-out pattern to reach their final destinations [23, 24]. In contrast, cortical inhibitory interneurons primarily originate in the ventral forebrain, with the majority produced in the medial ganglionic eminence (MGE) [25]. Their precursors in the ventricular and subventricular zones (VZ/SVZ) undergo terminal differentiation to produce early neuroblasts, or pioneer interneurons, which begin tangential migration toward the neocortex (NCX) around E11.5, followed by radial migration into specific cortical layers during the first postnatal days [26–28]. This migration process is governed by a complex interplay of extrinsic guidance cues and intrinsic cell-autonomous mechanisms including the coordinated remodeling of the actin and microtubule cytoskeleton [28]. It has been demonstrated that prenatal disruptions can affect both the extrinsic and intrinsic factors that regulate interneuron migration [29–33]. However, the impact of DNA damage-inducing factors on interneuron migration during neurodevelopment remains unknown.

The differential DNA damage response in dorsal and ventral OPCs [15], and differences in cell cycle regulation of dorsal and ventral NPCs [34, 35], may suggest a distinct response to DNA damage, potentially contributing to neurodevelopmental disorders. This may be further linked to an imbalance between excitatory and inhibitory signaling, as prenatal disturbances have been associated with (epileptic) seizures [3, 4, 36, 37], indicating a potential difference in the vulnerability of dorsal versus ventral NPCs to such damage. In this study, irradiation is used as a tool to investigate the effects of embryonic DNA damage on dorsal NCX and ventral MGE-derived NPCs. Our findings provide novel evidence of a region-specific response to irradiation-induced DNA damage, including significant changes in intrinsic pathway signaling related to interneuron migration, enhancing our understanding of how early developmental insults impact brain development.

## Materials and methods

### Experimental model

#### Mouse housing and husbandry

All animal experiments were performed in accordance with the Belgian laboratory animal legislation and the European Communities Council Directive (2010/63/EU), and approved by the local ethical committees at SCK CEN (Medanex Clinic), Hasselt University and Vrije Universiteit Brussel. Animals were maintained in a specific pathogen-free animal facility under standard laboratory conditions with access to food and water *ad libitum* under a 12 h light/dark cycle.

Mice were bred by mating female and male mice during a 2-h time period at the start of the light phase (7.30 AM to 9.30 AM) to ensure synchronous timing of embryonic development. Females with a vaginal plug at the end of the mating period were considered pregnant and designated with embryonic day 0 (E0). Pregnant mice were sacrificed by cervical dislocation in the morning at E11, E12, E13 and E15 and embryos were dissected. Embryos from two to five different litters were used to minimize the influence of developmental timing.

#### Mouse models

C57Bl/6J mice were obtained from Janvier (Bio Services, Uden, The Netherlands). *Emx1*^*Cre/+*^_;_*Trp53*^*fl/fl*^ (p53 cKO NCX) and *Emx1*^*Cre/+*^_;_*Trp53*^*fl/+*^ (p53 WT NCX) were obtained by first breeding *Trp53*^*fl/fl*^ mice (The Jackson Laboratory, Stock No. 008462) to *Emx1*^*Cre/Cre*^ mice (The Jackson Laboratory, Stock No. 005628), followed by crossing the conditional heterozygous *Emx1*^*Cre/+*^_;_*Trp53*^*fl/+*^ with *Trp53*^*fl/fl*^ as described before [19]. Additionally, *Emx1*^*Cre/+*^_;_*Trp53*^*fl/fl*^ were crossed with *Trp53*^*fl/fl*^ to acquire a higher yield of mutants. Similarly, *Nkx2.1*^*Cre/+*^_;_*Trp53*^*fl/fl*^ (p53 cKO MGE) and *Nkx2.1*^*Cre/+*^_;_*Trp53*^*fl/+*^ (p53 WT MGE) were obtained by first breeding *Trp53*^*fl/fl*^ mice to *Nkx2.1*^*Cre/+*^ mice (The Jackson Laboratory, Stock No. 008661), followed by crossing the conditional heterozygous *Emx1*^*Cre/+*^_;_*Trp53*^*fl/+*^ with *Trp53*^*fl/fl*^ mice. To obtain a higher yield of mutants, *Nkx2.1*^*Cre/+*^_;_*Trp53*^*fl/fl*^ were crossed with *Trp53*^*fl/fl*^. *Nkx2.1* reporter mice (*Nkx2.1*^*eGFP/+*^) were obtained by crossing *Nkx2.1*^*Cre/+*^ mice with R26R-EGFP mice (The Jackson Laboratory, Stock No. 032037-JAX).

#### Primary cell culture

The prefrontal cortex (NCX) and medial ganglionic eminence (MGE) of the right hemisphere of C57Bl/6J mice at E13 were dissected and mechanically dissociated by gentle pipetting in Accutase (Stemcell Technologies, 07920). Primary mouse NPCs were cultured as monolayers onto Poly-D-Lysine (PDL) coated plates (Corning) in Dulbecco’s Modified Eagle Medium (DMEM)/F-12 (Gibco, 11330-032) supplemented with B-27 (1:50, Gibco, 17504-044), N-2 (1:100, Gibco, 17502-048), Recombinant Human Fibroblast Growth Factor-basic (FGF-2, 20 ng/ml, Peprotech, 100–18 C) and with or without Recombinant Murine Epidermal Growth Factor (EGF, 10 ng/ml, Peprotech, 315-09), respectively. Cells were passaged every 3 to 4 days (~ 80% confluent) by centrifugation at 300 x g for 3 min, followed by mechanical redissociation and replating as described above.

#### Irradiation procedure

Females with a vaginal plug at E0 were (sham-) irradiated at E11 as described previously [19]. Briefly, single-dose whole body irradiation (1 Gy) was performed using an X-strahl 320 kV (0.13 Gy/min, inherent filtration: 0.21 mmAl, additional filtration: 3.8 mmAl + 1.4 mm Cu + DAP, tube voltage: 250 kV, tube current: 12 mA, source distance: 100 cm, vertical beam orientation) in accordance to ISO 4037. Sham mice (0 Gy) underwent the same procedure but were not placed within the irradiation field. The same settings were used to irradiate (0 and 1 Gy) NCX and MGE NPCs.

## Method details

### Mice genotyping

Tail (embryos) or ear (weaned pups) biopsies were digested in 200 µl DirectPCR Lysis Reagent (Viagen Biotech, 102-T) with 0.2 mg/ml proteinase K (Fermentas, E00492) at 56 °C for at least 2 h or overnight shaking, followed by 45 min at 85 °C. 5 µl of the obtained DNA was used for each PCR reaction.

### Immunohistochemistry

Embryos (E11) or brains (E13, E15) were dissected and fixed with 4% paraformaldehyde (PFA, Sigma-Aldrich) overnight at 4 °C. P14 and P56 offspring were transcardially perfused with 0.9% NaCl and fixed with 4% PFA. Brains were dissected and fixed with 4% PFA overnight at 4 °C. All samples were washed three times in PBS and stored in 70% ethanol at 4 °C until further manipulations. Next, samples were processed with the Leica TP1020 tissue processor (Leica Biosystems) and embedded in paraffin (Leica Biosystems, 39602004). Brains were cut in 7 μm thick coronal sections on a microtome (Thermo Fisher, Microm HM 340 E) and mounted onto Superfrost Plus Adhesion Microscope Slides (Epredia, J1800AMNZ) coated with glycerin albumen (VWR, 361002Y).

Paraffin tissue sections were deparaffinized using xylene (VWR, 28975.291) and rehydrated in graded solutions of ethanol (VWR, 20905.296 and 83672.290). Antigen retrieval was performed by boiling in pH6 citrate buffer (Dako, S1699) and samples were incubated (3 × 5 min) in 0.3% Triton X-100 (Merck, 9002-93-1) permeabilization buffer before blocking with either 3% Bovine Serum Albumin (BSA) (Sigma, A-2153)/0.3% Triton X-100/PBS, Normal Goat Serum (ThermoFisher, 31873) in Tris-NaCl blocking buffer (1:5) or 0.4% fish skin gelatin (Merck, G7041)/0.2% Triton X-100/PBS for 1 h at RT. After blocking, sections were incubated with primary antibody in Tris-NaCl blocking buffer overnight at RT. The following primary antibodies were used: rabbit anti-53BP1 (1:1000, Novus Biologicals, NB100-304), rabbit anti-PH3 (1:500, Cell Signaling, 3377), rabbit anti-CC3 (1:100, Cell Signaling, 9661), rabbit anti-PP53 (1:200, R&D systems, AF1043), rabbit anti-PV (1:1000, Abcam, ab11427), mouse anti-SST (1:200, Santa Cruz Biotechnology, sc-74556), goat anti-GFP (1:300, Abcam, ab5450) and mouse anti-BrdU (1:300, Bio-rad, MCA2483). The sections were washed (3 × 5 min) in 0.1% Triton X-100/tris-buffered saline and incubated for 2 h at RT with the appropriate Alexa Fluor-488, -568 or Cy5 (1:200, Invitrogen) secondary antibody in Tris-NaCl blocking buffer. Sections were washed (3 × 5 min) and nuclei were counterstained with DAPI for 15 min at RT. Slides were mounted with Prolong Diamond Antifade Mountant (Molecular probes, p36962) and images were taken using 20x air objective (NA 0.8) on a Nikon Eclipse Ti-E inverted microscope.

### Immunocytochemistry

NCX and MGE NPCs were seeded on PDL-coated 96-well plates (Greiner, 655946) or PDL-coated coverslips (Corning, 354086) at least 24 h before staining. Cells were fixed with 4% PFA for 15 min at RT, washed (3 × 5 min) in PBS and permeabilized with 0.1% Triton X-100/3% BSA/PBS for 5 min at RT. Blocking was performed using either 5% BSA/PBS or 5% Normal Goat Serum/PBS for 1 h at RT and incubated with primary antibodies diluted in blocking buffer overnight at 4 °C. The following primary antibodies and respective dilutions were used: mouse anti-γH2AX (1:200, Merck Millipore, 05-636), rabbit anti-53BP1 (1:1000, Novus Biologicals, NB100-304), rabbit anti-PH3 (1:500, Cell Signaling, 3377), rabbit anti-CC3 (1:200, Cell Signaling, 9661), mouse anti-Emx1 (1:300, Santa Cruz, sc-398115), rabbit anti-TTF1 (Nkx2.1, 1:250, Abcam, ab76013), mouse anti-Ascl1 (1:500, Santa Cruz, sc-374104), rabbit anti-Pax6 (1:300, BioLegend, 901301) and mouse anti-Nestin (1:300, Invitrogen, MA1-110). Cells were washed (3 × 5 min) in TBS and incubated with Alexa Fluor-488, -568 or Cy5 secondary antibodies for 2 h at RT. Cells were then washed (3 × 5 min) in TBS and mounted with ibidi mounting medium with DAPI (Ibidi, 50011) or Prolong Diamond Antifade Mountant (Molecular probes, p36962). Images are either acquired using a 20x air objective (NA 0.8) on a Nikon Eclipse Ti-E inverted microscope or using a 20x Plan Fluorite Objective (Olympus, NA 0.45) on a BioTek Citation 5 microscope (Agilent Technologies).

### Image analysis

All images were analyzed using ImageJ. For each biological replicate, defined as an individual embryo or P56 mouse, one to seven tissue sections were analyzed. The dorsal NCX and/or MGE from both the left and right brain hemispheres were included in the analysis. The InteractiveCountTools_subregio in ImageJ was used to delineate the dorsal NCX and MGE in E11 brain slices. To quantify SST + and PV + cells in P56 brains, the hippocampus and somatosensory cortex were manually demarcated using The Allen Mouse Brain Atlas [38], with the latter being divided into cortical layers I to VI. For NPC cultures, two to three biological replicates (cells derived from different embryos) were analyzed. Here, the CellBlock plugin in ImageJ was used to analyze γH2AX and 53BP1 spots, PH3-, CC3-, Pax6-, Emx1-, Nkx2.1-, Ascl1- and DAPI-positive cells [39, 40].

PH3 cells in late G2 and M phase were counted either manually or using the InteractiveCountTools_subregio in ImageJ in the apical and basal zone of the NCX and MGE. The apical zone corresponds to progenitors in the VZ (i.e. in proximity of the ventricles), the basal zone corresponds to progenitors in the SVZ or in proximity of the CP. In late G2, cells display a speckled appearance of PH3 staining while in M phase, the cells display pan-nuclear PH3 staining [41].

### Interneuron migration in fixed brain slices (BrdU pulse labeling)

Pregnant *Nkx2.1*^*eGFP/+*^ mice (E11) received intraperitoneal injections of 5-bromo-2’-deoxyuridine (BrdU, 50 mg/kg body weight, Sigma-Aldrich) 30 min before irradiation. Embryonic brains were harvested at E13 and E15 and processed as mentioned above. Genotyping was performed to select *Nkx2.1*^*eGFP/+*^ offspring. Cell counting was performed in boxes spanning 150 μm of the P-SP boundary or NCX using ImageJ. As irradiated embryonic brains were smaller than non-irradiated embryonic brains, the amount of GFP^+^/BrdU^+^ and GFP^+^/BrdU^−^ cells were corrected for the reduction in the amount of DAPI-positive cells.

### Interneuron migration in acute living embryonic brain slices using two-photon imaging

Embryonic brains from E13.5 *Nkx2.1*^*eGFP/+*^ mice were isolated in ice-cold PBS-glucose (pH 7.4, 25 mM glucose) and embedded in 4% low melting agarose (Thermo Fisher Scientific, 16520100). Prior to slicing, genotyping was performed to select *Nkx2.1*^*eGFP/+*^ offspring. 300 μm thick coronal slices were made using a vibratome (Leica VT1200S). Brain slices were transferred to MilliCell organotypic inserts (8 μm pores, Merck Millipore) in a 24-well plate designed for confocal microscopy (IBIDI) and maintained in semi-hydrous conditions, at 37 °C and 5% CO_2_. Migration medium consisted of Neurobasal medium (Thermo Fisher Scientific, 12348017) supplemented with 2 mM L-glutamine (Thermo Fisher Scientific, 25030081), B-27 supplement (Thermo Fisher Scientific, 17504-044), N2 supplement (Thermo Fisher Scientific, 17502-048) and 0.5% P/S (Thermo Fisher Scientific, 15140130).

Image acquisition started 1 h after tissue equilibration at 37 °C and 5% CO_2_, and was completed within 8 h after brain dissection. During measurements, the environment chamber of the microscope was heated by constant air provision, at 37 °C, and 5% CO2 humidified air was applied directly onto the slice. A confocal microscope (ZEISS LSM880) provided with a Mai Tai DeepSee Ti: Sapphire pulsed laser (Spectra-Physics) tuned at 780 nm and a 20x EC plan-Neofluar objective (NA of 0.5 and 2 mm working distance) was used to image EGFP-labeled interneurons in the embryonic brain. A z-stack spanning 72 μm (8 μm interval) with serial optical sections at 1024 × 1024 pixels was acquired every 10 min for a total duration of 6 h. Stacks were recorded starting from a minimum depth of 50 μm beneath the surface of the slice.

### Interneuron migration in MGE explants

MGE explants were dissected from E12 brains in ice-cold dissection medium containing Neurobasal medium (Thermo Fisher Scientific, 21103049) supplemented with 2 mM L-glutamine (Thermo Fisher Scientific, 25030024), B-27 supplement (Thermo Fisher Scientific, 17504-044), N2 supplement (Thermo Fisher Scientific, 17502-048) and 0.5% P/S (Sigma-Aldrich, P4333). Dissected MGE was cut in 3 pieces (explants) and each explant was pipetted onto a PDL-coated 96-well plate (Greiner, 655946) containing ice-cold 0.3% methylcellulose (Sigma-Aldrich, M0512) in neurobasal medium (Thermo Fisher Scientific, 21103049) supplemented with 2 mM L-glutamine (Thermo Fisher Scientific, 25030024), B-27 supplement (Thermo Fisher Scientific, 17504-044), N2 supplement (Thermo Fisher Scientific, 17502-048) and 0.5% P/S (Sigma-Aldrich, P4333). Explants were able to stabilize for at least 1 h at 37 °C and 5% CO_2_.

After 4–6 h in culture, MGE explants were imaged using a BioTek Citation 5 microscope (Agilent Technologies) with a BioTek BioSpa 8 Automated Incubator (Agilent Technologies) to ensure controlled temperature and CO_2_ conditions. A z-stack spanning 20 μm (4 μm interval) was taken every 30 min for a total of 48 h using a 10x Plan Fluorite Objective (Olympus, NA 0.3).

### Analysis of interneuron migration

Time series were corrected for 3D drift using the ImageJ Correct 3D drift or StackReg plugin and interneuron migration was manually tracked using the MTrackJ plugin. Average migration speed (µm/h) was calculated as the total length of the migration path divided by the duration of the track. The instantaneous speed was calculated using Excel macro Phase Durations and MSD [42, 43]. The threshold for idling was set at half a cell diameter (8 μm) per 10 min, and confirmed by using a custom-made macro developed by Gorelik and Gautreau [42]. Instantaneous speeds of active migration events are defined as events above the idling threshold of 0.8 μm/min and were calculated as the distance traveled between each time frame, divided by the frame interval. The number of jumps are defined as the amount of events larger than the average cell diameter (± 15 μm) in MGE explants, divided by the time.

### Acute seizure induction

Male P56 offspring received continuous infusion of 7.5 mg/ml of PTZ (Sigma Chemical Company) with 5000 IE/ml heparin dissolved in 0.9% NaCl solution as described before [44]. Infusion was performed using a 29-G needle, attached to polyethylene tubing (Smiths) and inserted into the tail vein at a constant rate of 150 µl/min, using a Hamilton syringe mounted to an infusion pump (CMA, Microdialysis). Animals were allowed to move freely in a plexiglas cage. The threshold for the different phases of PTZ-induced seizure activity was determined using the following endpoints: (1) ear twitch, (2) myoclonic twitch, (3) forelimb clonus, (4) clonus with loss of righting reflexes (falling), (5) tonic hind limb extension (THE), and (6) death. Time was measured from the start of PTZ infusion until the onset of these endpoints. The seizure threshold was determined for each animal individually according to the following equation: dose (mg/kg) = duration of infusion (s) x rate of infusion (ml/min) x drug concentration (mg/ml) x 1000/(60 s x weight of mouse (g)).

### Quantitative reverse transcriptase PCR (qRT-PCR)

NCX and MGE NPCs were lysed in RLT Plus lysis buffer (Qiagen, 74136) and samples were stored at -80 °C until further processing. RNA was extracted using the RNeasy Mini or Micro kit (Qiagen, 74136) and eluted in 30 µl RNase-free water. Next, cDNA was synthesized using the GoScript Reverse Transcriptase kit (Promega, A2801). qRT-PCR was performed using a qTOWER^3^G (Analytik Jena) and the QuantiNova SYBR Green PCR kit (Qiagen, 208252). Relative gene expression was calculated using *Polr2a* as reference gene. The list of primers can be found in Table 1.

**Table 1:** List of primers used for qRT-PCR

	Forward (5’ ◊ 3’)	Reverse (5’ ◊ 3’)
*Polr2a*	GCACCACGTCCAATGATATTGTG	GGAGATGACATGGTACAGTTCTCG
*Hes5*	GGAGATGCTCAGTCCCAAGG	GCTCTATGCTGCTGTTGATGC
*Pax6*	ACACGTACAGTGCTTTGCCA	GCAGCATGCACGAGTATGAG
*Nkx2.1*	TGTCCTCGGAAAGACAGCAT	GAGAACGGAGTCGTGTGCTT
*Ascl1*	ACTTGAACTCTATGGCGGGTT	CCAGTTGGTAAAGTCCAGCAG
*Emx1*	TGGAGCGAGCCTTTGAGAAG	GGAACCACACCTTCACCTGC

### Western blotting

NCX and MGE NPCs were lysed in cold RIPA lysis and extraction buffer (ThermoFisher, 89901) with Halt™ Protease and Phosphatase Inhibitor Cocktail (ThermoFisher, 78440). Proteins were collected by aspirating the supernatant after centrifugation of the cell lysate. Protein concentration was determined using the bicinchoninic acid protein (BCA) assay kit (Bio-Rad, 5000201). Protein samples diluted in Laemmli buffer with 10% β-mercaptoethanol were heated for 5 min at 95 °C and used for SDS-PAGE with a 4–20% Criterion™ TGX Stain-Free™ Protein Gel (Bio-Rad, 5678093). Gels were transferred using a Trans-Blot Turbo Midi PVDF Transfer Package (Bio-Rad, 1704157) and PVDF membranes were blocked for 1–2 h with 3% BSA/0.1% TBS-Tween20 (ThermoFisher, J60497.K3) blocking buffer. Primary antibodies diluted in blocking buffer were incubated overnight at 4 °C. The following primary antibodies were used: rabbit anti-Dcx (1:1000, abcam ab18723), mouse anti-Akt (1:1000, cell signaling, 2920), rabbit anti-pAkt (1:1000, cell signaling, 9271), rabbit anti-Erk1/2 (1:1000, cell signaling, 4695), rabbit anti-pErk1/2 (1:1000, cell signaling, 4370), rabbit anti-Cofilin (1:1000, cell signaling, 3318), rabbit anti-pCofilin (1:1000, cell signaling, 3311), mouse anti-αPak (1:100, Santa Cruz, sc-166887), rabbit anti-pPak1/2 (1:800, cell signaling, 2605), and mouse anti-GAPDH (1:4000, abcam, ab8245). The membranes were washed (3 × 10 min) in 0.1% TBS-Tween20 and incubated for 1 h at RT with HRP-conjugated secondary antibodies (goat anti-rabbit (ThermoFisher, 65-6120) or goat anti-mouse (ThermoFisher, 62-6520)) diluted in blocking buffer. Proteins were visualized using the enhanced chemiluminescence system (ECL) (ThermoFisher, A38555) and Amersham ImageQuant 800 Western blot imaging system (Cytiva). Analysis was performed using the ImageQuantTL v10.2 software (Cytiva).

### Sample collection and cDNA library construction for RNA sequencing

NCX and MGE NPCs were lysed at 6 and 24 h following irradiation in RLT Plus lysis buffer (Qiagen, 74136) and samples were stored at -80 °C until further processing. RNA was extracted using the RNeasy Mini kit (Qiagen, 74136) according to the manufacturer’s protocol. cDNA library construction and sequencing were performed by NovoGene (Cambridge, United Kingdom). Messenger RNA (mRNA) was purified from total RNA using poly-T oligo-attached magnetic beads. Library quality was assessed using Qubit, real-time PCR for quantification, and a bioanalyzer for size distribution. Quantified libraries were pooled and sequenced using paired-end 150 bp reads (PE150) on an Illumina sequencing platform.

### RNA sequencing data and differential expression (DE) analysis

RNA sequencing data were preprocessed with nf-core/rnaseq version 3.12.0 [45] using the default parameters except for: (i) NCBI GRCm39 (NCBI RefSeq assembly: GCF_000001635.27) was used as the reference genome, (ii) salmon quant was run with the “--gcBias” parameter. Differential expression analysis was performed with nf-core/differentialabundance version 1.4.0 [45] with the default values. For GSEA, M2 curated gene sets from the Mouse MSigDB Collections was used. For further analysis, only genes with a false discovery rate (FDR) of less than 0.05 were considered statistically significant and included in downstream analyses.

### Enrichment analysis

To assess overrepresented biological processes, pathways or transcription factor binding sites, the Enrichr platform (https://maayanlab.cloud/Enrichr/) was used. The ‘GO Biological Process 2023’ ‘KEGG 2021’ and ‘ENCODE and ChEA Consensus TFs from ChIP-X’ datasets were employed for enrichment analysis.

### Statistical analysis

Statistical analyses was performed using GraphPad Prism 10. Data distributions were evaluated for normality using the Shapiro Wilk test. If normality was confirmed for all groups, a one-tailed Student’s t-test or One-Way ANOVA test followed by Tukey’s test for multiple comparisons was performed when comparing between two or more conditions, respectively. If at least one group exhibited a non-Gaussian distribution, non-parametric tests were used instead, such as Mann-Whitney U test for two groups or Kruskal-Wallis test followed by Dunn’s post-hoc test for multiple comparisons. If standard deviations were significantly different, Welch ANOVA test followed with Dunnett’s test for multiple comparisons was used. For primary immunostainings, qRT-PCR and western blot results of NPC culture data, paired equivalents of the aforementioned tests were utilized. P-values smaller than 0.05 were considered significant with * *p* < 0.03, ** *p* < 0.002, *** *p* < 0.0002 and **** *p* < 0.0001. Data is represented as mean ± SD. In all figure legends, sample size n represents the number of embryos, P56 mice, or biological replicates of NPCs. Sample size N represents the number of individual cells used for analysis. Additional statistical details are provided in the figure legends.

## Results

### Prenatal irradiation triggers distinct and overlapping DNA damage responses in the NCX and MGE

Based on significant reductions in body and brain weight with minimal offspring mortality (Fig. S1), the experimental conditions for prenatal irradiation exposure were determined to be 1 Gy irradiation at E11 and used in all experiments.

**Fig. 1:**
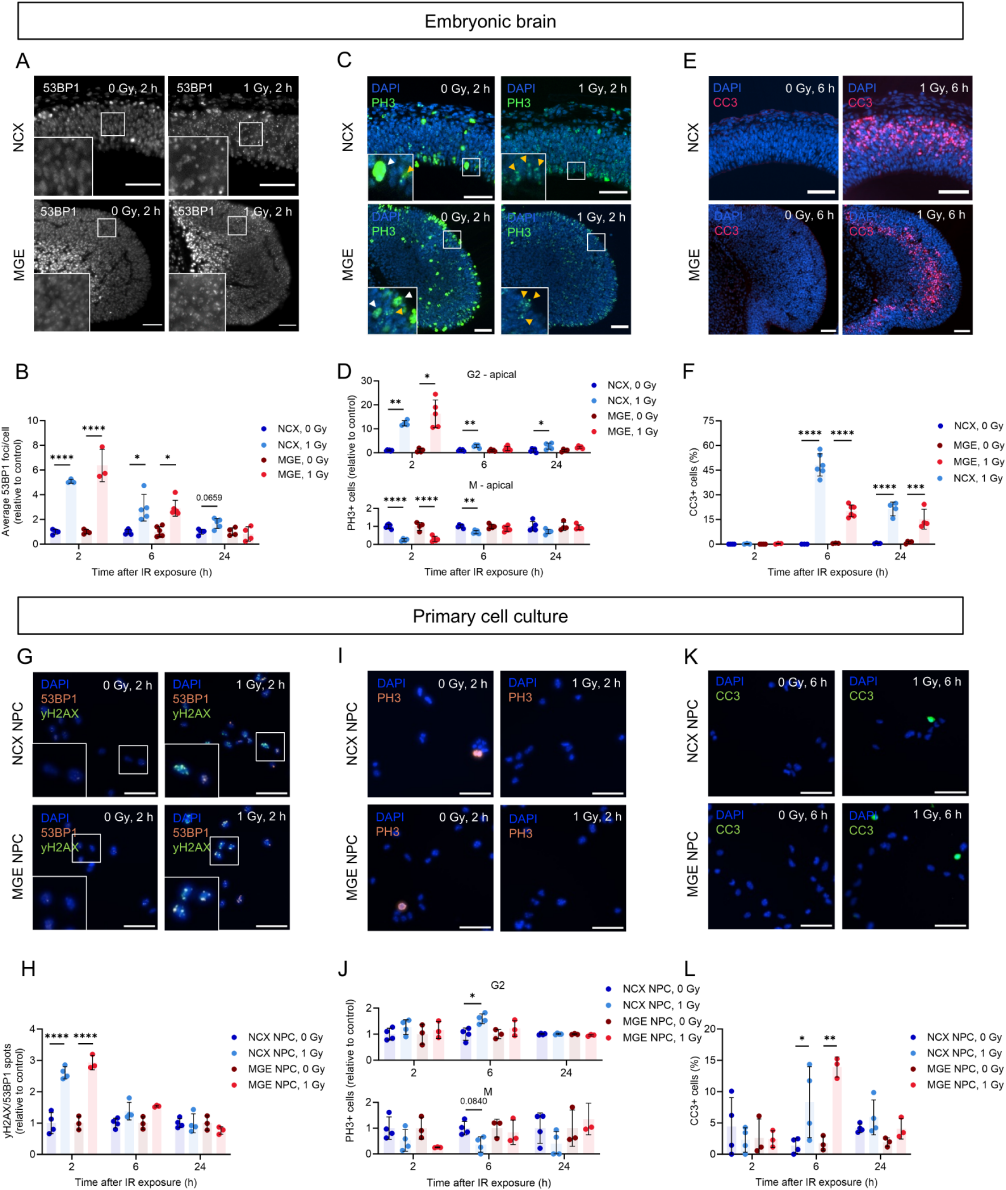
Prenatal irradiation triggers distinct and overlapping DNA damage responses in the NCX and MGE. (**A**,** C**,** E**) Immunostaining of DSB marker 53BP1 (**A**), late G2/M phase marker PH3 (**C**) and apoptosis marker CC3 (**E**) in NCX and MGE, 2 h (**A**, **C**) or 6 h (**E**) post-irradiation. (**C**) White arrowheads mark examples of cells in the M phase, while yellow arrowheads mark examples of cells in the late G2 phase. (**B**,** D**,** F**) Average 53BP1 foci/cell (**B**) and PH3-positive cells (G2/M) normalized to control (**D**), and percentage CC3-positive cells (**F**) in NCX and MGE of embryonic brains. *n* = 3–6. (**G**,** I**,** K**) Immunostaining of DSB markers 53BP1 and γH2AX (**G**), late G2/M phase marker PH3 (**I**) and apoptosis marker CC3 (**K**) in NCX and MGE primary cell cultures, 2 h (**G**, **I**) or 6 h (**K**) post-irradiation. (**H**,** J**,** L**) 53BP1/γH2AX spots/cell (**H**), PH3-positive cells (G2/M) (**J**) and CC3-positive cells (**L**) all relative to control, in NCX and MGE primary cell cultures. NCX NPCs_0 Gy: *n/N*_53BP1/γH2AX_ = 4/320 (2 h), 4/414 (6 h), 4/259 (24 h); *n/N*_PH3_ = 4/425 (2 h), 4/298 (6 h), 4/569 (24 h); *n/N*_CC3_ = 4/529 (2 h), 4/394 (6 h), 4/372 (24 h); NCX NPCs_1 Gy: *n/N*_53BP1/γH2AX_ = 4/486 (2 h), 4/267 (6 h), 4/167 (24 h); *n/N*_PH3_ = 4/763 (2 h), 4/383 (6 h), 4/407 (24 h); *n/N*_CC3_ = 4/484 (2 h), 4/307 (6 h), 4/703 (24 h); MGE NPCs_0 Gy: *n/N*_53BP1/γH2AX_ = 3/1081 (2 h), 3/1958 (6 h), 3/1088 (24 h); *n/N*_PH3_ = 3/1216 (2 h), 3/1340 (6 h), 3/1683 (24 h); *n/N*_CC3_ = 3/1143 (2 h), 3/2085 (6 h), 3/1567 (24 h); and MGE NPCs_1 Gy: *n/N*_53BP1/γH2AX_ = 3/1287 (2 h), 3/782 (6 h), 3/923 (24 h); *n/N*_PH3_ = 3/1208 (2 h), 3/1393 (6 h), 3/769 (24 h); *n/N*_CC3_ = 3/1327 (2 h), 3/1085 (6 h), 3/1049 (24 h). One-way ANOVA test followed by Tukey’s test for multiple comparisons, Kruskal-Wallis with Dunn’s test for multiple comparisons or Welch ANOVA test followed with Dunnett’s test for multiple comparisons was used. Scale bar = 50 μm

To validate the DNA damage response following irradiation exposure and investigate potential variations in the response between cells in the NCX and MGE, we evaluated DNA damage and DNA damage response markers in both brain regions at 2, 6 and 24 h post-irradiation. Irradiation-induced DNA damage often presents as DNA DSBs, the most lethal form of DNA damage [46]. Therefore, DSBs were assessed by immunostainings for 53BP1, which localizes to DSBs to activate DNA repair and cell cycle signaling (Fig. 1A; Fig. S2A, B). A strong increase in the number of DSB foci per cell was observed at 2 h post-irradiation which returned to baseline levels within 24 h in both the MGE and NCX (Fig. 1B). This indicates that irradiation exposure leads to DNA, which is repaired in both regions, provided the cells have not sustained severe damage.

P53 safeguards the genome by orchestrating various DNA damage response mechanisms, including facilitation of DNA repair by inducing cell cycle arrest, allowing sufficient time for DNA repair mechanisms to repair the damage before the cell cycle proceeds [14, 47]. To evaluate the cell cycle dynamics following irradiation, we stained for phospho-histone 3 (PH3), a marker for cells in late G2 and mitosis (M) [41] (Fig. 1C; Fig. S2C, D). We separately quantified cells in G2 phase (speckled staining) and in M phase (pan-nuclear staining). This showed an increase in G2 cells similar to findings in neuro-epithelial cells that exhibited a DNA damage-like response due to *Cdh1* deficiency [48]. More specifically, in the apical zone, where most dividing early-born NPCs are located [49], an increase in cells in late G2 and a decrease in cells in the M phase was observed in both the NCX and MGE at 2 h post-irradiation (Fig. 1D), indicating a G2/M cell cycle arrest. In the MGE, this cell cycle arrest had resolved at 6 and 24 h post-irradiation, while a partial G2/M arrest was still evident in the NCX at these timepoints (Fig. 1D). In the basal zone, where more differentiated progenitors are typically found [49], fewer cells underwent a G2/M arrest at 2 h post-irradiation which had largely resolved by 6 h in both the NCX and MGE (Fig. S2E). Notably, a larger increase in G2 phase cells was seen in the MGE compared to the NCX at 2 and 24 h post-irradiation (Fig. S2E). These results confirm the induction of a DNA damage response in the NCX and MGE upon irradiation and showed a prolonged cell cycle arrest in the NCX in comparison to the MGE.

Although many cells exhibited efficient DNA repair, p53 can induce apoptosis in cells in which DNA repair was not successful [14, 47]. Therefore, the apoptotic marker cleaved-caspase 3 (CC3) was used to evaluate irradiation-induced apoptosis (Fig. 1E; Fig.S2F, G). A significant increase in apoptotic cells was observed 6 and 24 h post-irradiation in both the NCX and MGE (Fig. 1F). At 6 h post-irradiation, approximately 48% of cells in the NCX and 20% in the MGE were apoptotic. By 24 h, this dropped to about 21% in the NCX and 15% in the MGE (Fig. 1F). This reflects a 2.2-fold decrease in apoptotic cells in the NCX, compared to a smaller 1.3-fold decrease in the MGE, indicating more sustained apoptosis over time in the MGE compared to the NCX. Collectively, our results demonstrate that DNA repair, cell cycle arrest, and apoptosis are triggered in both the NCX and MGE following irradiation, indicating a typical DNA damage response. Interestingly, we also observed differences between these regions, suggesting cell type-specific DNA damage responses, with the NCX showing prolonged cell cycle arrest and more pronounced decline in apoptosis compared to the MGE.

To further investigate potential differences in the DNA damage response specifically between the proliferative NPCs of the NCX and MGE, primary NPC cultures were established from these brain regions. This approach allowed both the study of the cell-autonomous response in a controlled environment and a direct comparison of the DNA damage response between the two cell types. Briefly, NCX and MGE primary cell cultures were derived from prefrontal NCX and MGE of E13 brains and cell cultures were evaluated 2, 6 and 24 h post-irradiation (0 and 1 Gy), using immunostainings for the same markers as described above. First, the lineage of NCX and MGE cultures was confirmed by the expression of dorsal (Pax6 and Emx1) and ventral (Nkx2.1 and Hes5) cell markers in NCX and MGE cultures, respectively (Fig.S3). Ascl1 was expressed in both cell types, albeit at higher levels in MGE-derived cells (Fig. S3) [50]. Furthermore, the NPC identity in both cultures was validated by the expression of the NPC-specific marker Nestin (Fig. S3A). An increase in yH2AX/53BP1 foci, indicating DNA DSB damage, was found 2 h post-irradiation in both NCX and MGE cultures and returned back to baseline levels over time (Fig. 1G, H; Fig. S2H, I). An increase in G2-cells was only observed 6 h post-irradiation in NCX cultures, and a decreasing trend in M-phase cells was observed 2, 6 and 24 h post-irradiation in NCX cultures, while in MGE cultures this trend was only evident at 2 h (Fig. 1I, J; Fig. S2J, K). This shows that 6 h post-irradiation a G2/M cell cycle arrest is present in the NCX. Although only a reducing trend in M-phase cells was observed at 2 and 24 h, these results suggest a G2/M cell cycle arrest at 2 h post-irradiation, which resolved by 6 h in MGE but not in NCX cultures. Additionally, an increase in apoptotic CC3-positive cells was observed 6 h post-irradiation in both NCX and MGE cultures (Fig. 1K, L; Fig. S2L, M). Overall, the DNA damage response in primary NCX and MGE cell cultures are in line with our observations in the embryonic brain, indicating a prolonged cell cycle arrest in the NCX.

### P53 governs the DNA damage response in the NCX and MGE

As previously mentioned, the tumor suppressor protein p53 is well-known for its critical role in the DNA damage response, particularly in DNA damage-induced cell cycle arrest and apoptosis [14, 47, 51]. To investigate the specific involvement of p53 in the radiation response of the NCX and MGE, *Trp53* conditional knockout (cKO) mice were generated. Specifically, *Trp53* was conditionally knocked out in the neurons of the dorsal forebrain (*Emx1*^*Cre/+*^_;_*Trp53*^*fl/fl*^, referred to as cKO NCX) [19] and in MGE-derived telencephalic interneurons (*Nkx2-1*^*Cre/+*^_;_*Trp53*^*fl/fl*^, referred to as cKO MGE) (Fig.S4A). Control groups consisted of *Trp53*^*fl/fl*^ littermates from both breedings (WT NCX and WT MGE). The conditional knockout of *Trp53* in the MGE was validated by PCR and immunostaining for phosphorylated p53 (p-p53) (Fig.S4B, C). Mfossa et al. confirmed the absence of *Trp53* expression in the dorsal forebrain of cKO NCX mice [19].

**Fig. 2:**
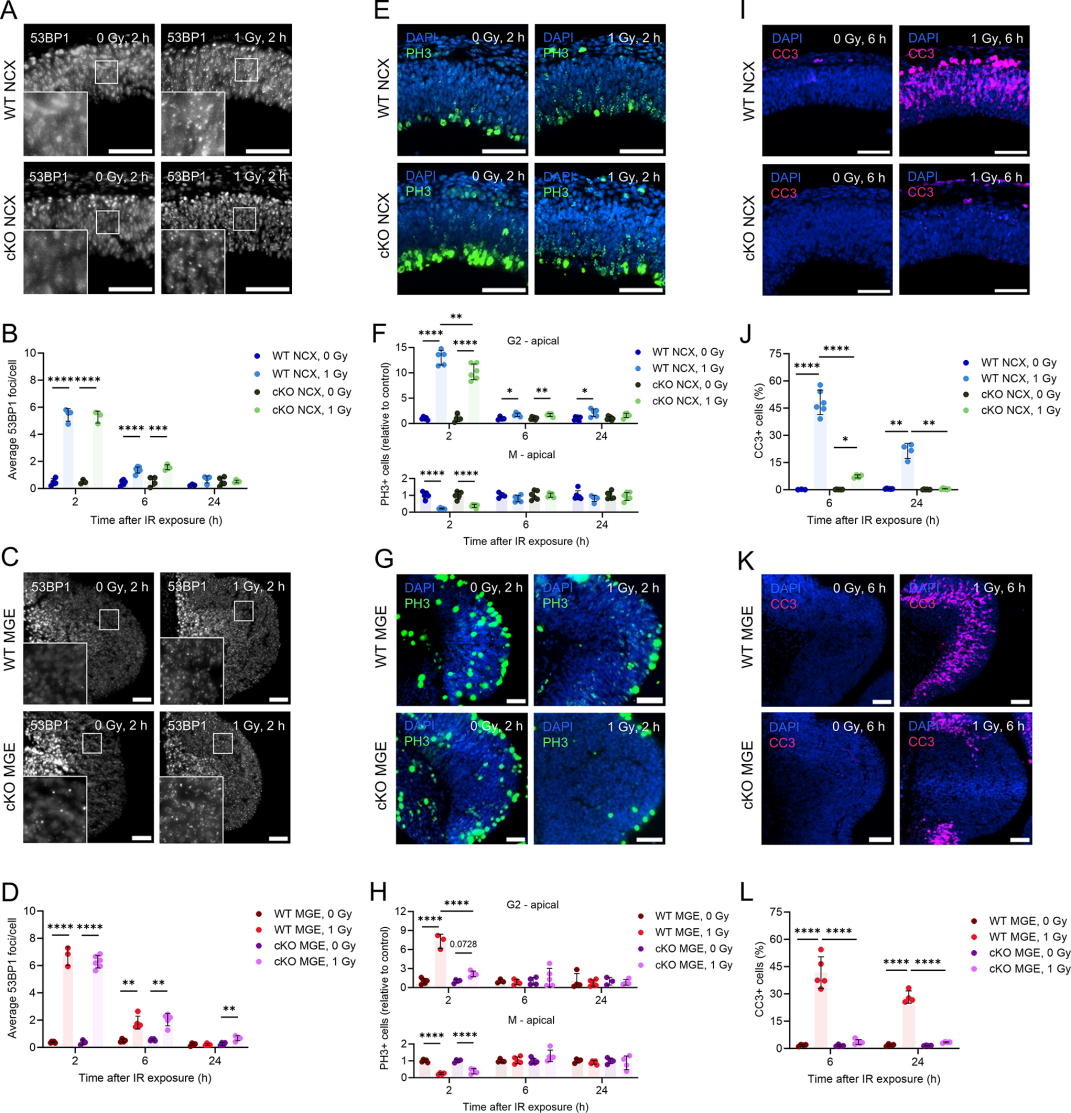
P53 governs the DNA damage response in the NCX and MGE. (**A**,** C**) Immunostaining of DSB marker 53BP1. Representative images of NCX and MGE of WT, cKO NCX and cKO MGE, 2 h post-irradiation at E11. (**B**,** D**) Average 53BP1 foci/cell in the NCX of WT and p53 cKO NCX embryonic brains (**B**) and MGE of WT and p53 cKO MGE embryonic brains (**D**). (**E**,** G**) Immunostaining of late G2/M phase marker PH3. Representative images of NCX and MGE of WT, cKO NCX and cKO MGE, 2 h post-irradiation at E11. (**F**,** H**) Relative amount of PH3-positive cells in G2- and M-phase (relative to control) in the apical zone of NCX of WT and p53 cKO NCX embryonic brains (**F**) and MGE of WT and p53 cKO MGE embryonic brains (**H**). (**I**,** K**) Immunostaining of apoptosis marker CC3. Representative images of NCX and MGE of WT, cKO NCX and cKO MGE, 2 h post-irradiation at E11. (**J**,** L**) Percentage CC3-positive cells in the NCX of WT and p53 cKO NCX embryonic brains (**J**) and MGE of WT and p53 cKO MGE embryonic brains (**L**). *n* = 3–6. One-way ANOVA test followed by Tukey’s test for multiple comparisons, Kruskal-Wallis with Dunn’s test for multiple comparisons or Welch ANOVA test followed with Dunnett’s test for multiple comparisons was used. Scale bar = 50 μm

The genetic ablation of *Trp53* in both the NCX and MGE did not substantially affect the number of DSB foci or DNA repair, as the foci levels returned nearly back to baseline across all conditions (Fig. 2A-D; Fig. S5A-D). However, in line with the role of P53 to initiate cell cycle arrest [14, 47], cell cycle arrest was less efficient in irradiated cKO NCX and cKO MGE mice as evidenced by a reduced number of apical G2-phase cells compared to irradiated WT mice at 2 h post-irradiation, especially in the MGE (Fig. 2E-H). No differences were observed in the numbers of apical M-phase, and basal G2- and M-phase cells between irradiated WT and cKO mice (Fig. 2E-H; Fig. S5E-J). At 6 and 24 h post-irradiation, the cell cycle arrest was resolved in the MGE while the prolonged G2/M arrest in the NCX could be confirmed (Fig. 2E-H, Fig. S5G-J). Lastly, we evaluated radiation-induced apoptosis in cKO mice. We found a dramatic reduction in the number of apoptotic cells in irradiated cKO mice compared to their WT littermates at both 6 and 24 h post-irradiation in both the NCX and MGE (Fig. 2I-L; Fig.S5K-L). Thus, p53 was found to be essential for cell cycle arrest and apoptosis following prenatal irradiation in both the NCX and MGE.

### Transcriptional profiling confirms a p53-dependent DNA damage response and uncovers divergent patterns of cell cycle arrest in NCX and MGE NPCs

**Fig. 3:**
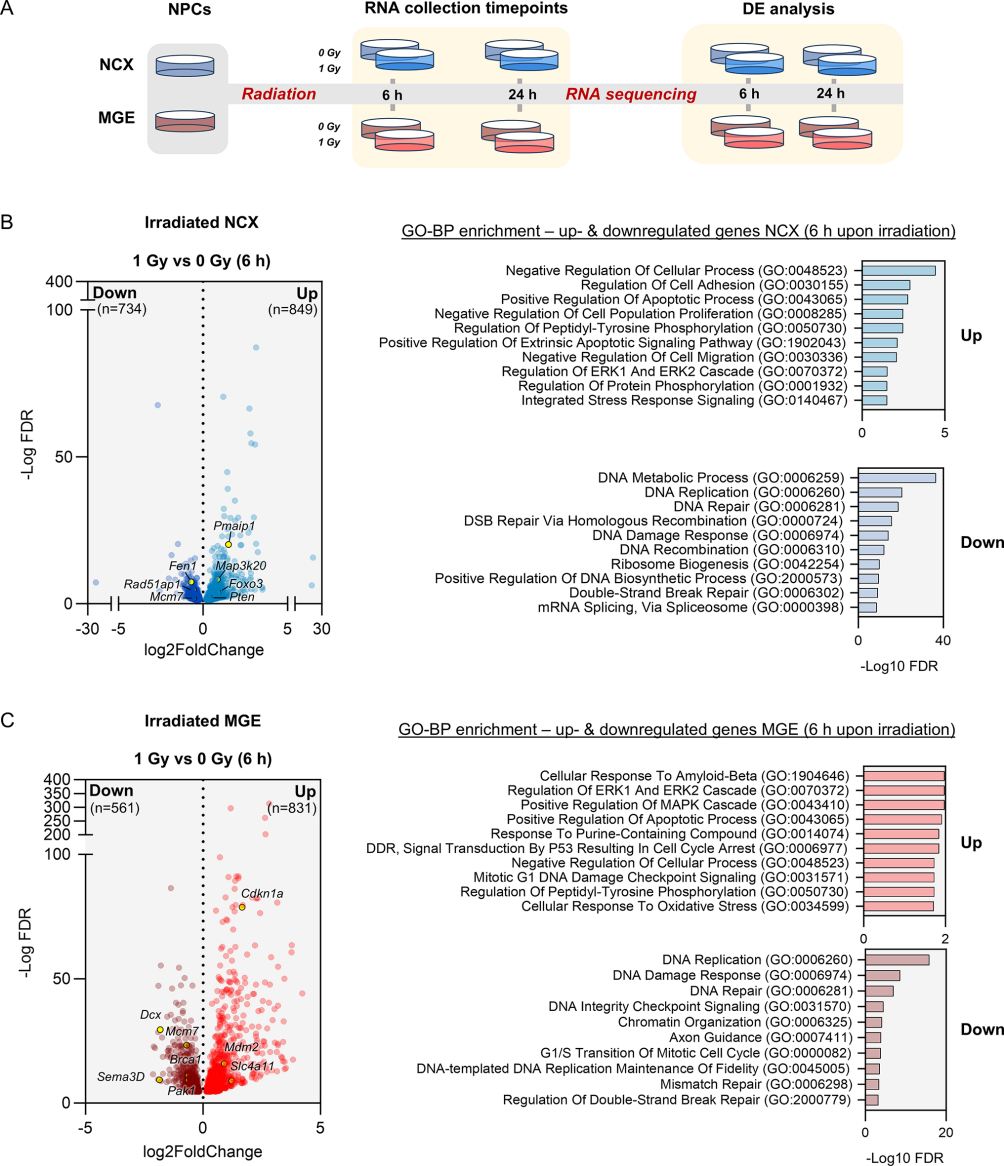
Transcriptional profiling confirms a p53-dependent DNA damage response in MGE and NCX NPCs, 6 h post-irradiation. (**A**) Schematic illustration of the RNA-seq experimental design. Primary NCX and MGE cultures were exposed to 0 (sham) or 1 Gy of radiation. RNA was extracted at 6 and 24 h post-irradiation, and RNA-seq data were analyzed for differential gene expression (DE) across conditions. (**B**) (Left) Volcano plot of significant DEGs (FDR < 0.05) between 1 Gy irradiated and sham (0 Gy) NCX NPCs at 6 h post-irradiation. Upregulated DEGs in NCX are shown in light blue (*n* = 849), while downregulated DEGs are shown in dark blue (*n* = 734). (Right) Enrichment analysis of GO BP terms for upregulated and downregulated NCX DEGs 6 h post-irradiation, with relevant DEGs highlighted in the volcano plot on the left. (**C**) (Left) Volcano plot showing significantly upregulated (*n* = 831, light red) and downregulated (*n* = 561, dark red) DEGs (FDR < 0.05) between 1 Gy irradiated and sham (0 Gy) MGE NPCs at 6 h. (Right) Enrichment analysis of GO BP terms associated with upregulated and downregulated MGE DEGs 6 h post-irradiation. Relevant DEGs are highlighted in the volcano plot on the left

To further elucidate potential differences in the impact of DNA damage on NCX- and MGE-derived NPCs, RNA-seq was performed. RNA was extracted from NCX and MGE NPCs at 6 and 24 h post-irradiation (Fig. 3A; Table S1). As a further confirmation of the purity of our cultures, we first compared untreated NCX and MGE NPCs. This showed that enormous differences exist in gene expression between these cell types, with no less than 8,790 genes being differentially expressed (DE), equally divided between up- and downregulated genes (Fig. S6A; Table S1). Among these, several dorsal and ventral cell markers were observed, further highlighting the distinct identity and origin of these cell types (Fig. S6A). Gene Ontology (GO) enrichment analysis revealed that NCX cells exhibited enrichment in genes related to ribosome biogenesis, gene expression, mRNA splicing via spliceosome, as well as mitochondrial gene expression, and mitochondrial translation (Fig. S6A). In contrast, MGE NPCs were predominantly enriched in processes related to nervous system development, including synapse organization, axon guidance, and synaptic signaling (Fig. S6A).

To investigate potential differences in the transcriptional response of NCX and MGE NPCs to irradiation, we investigated gene expression changes at both 6 h and 24 h post-irradiation. After 6 h, both NCX and MGE NPCs displayed a robust upregulation of processes associated with cellular stress responses, particularly those related to cell cycle regulation, apoptosis and signal transduction (Fig. 3B, C; Figs. S7; S8A; Table S1). Importantly, the p53 signaling pathway was prominently upregulated in both cell types, as evidenced by GO-BP, KEGG and MSigDB hallmarks (Fig. 3C; Fig.S7; Fig. S8A) underscoring its critical role in mediating the cellular response to DNA damage. Furthermore, both NCX and MGE NPCs showed significant enrichment for p53 as one of the top transcription factors for upregulated DEGs at 6 h post-irradiation (Fig. S7B). The PI3K-Akt and MAPK signaling pathways, crucial in roles such as cell survival, proliferation and stress response [52–55], were also upregulated in both cell types at 6 h post-irradiation (Fig. S7A). Both NCX and MGE NPCs at 6 h post-irradiation exhibited downregulation of key processes involved in DNA replication, repair (e.g. mismatch repair and homologous recombination) and cell cycle progression, as observed in both GO-BP and KEGG analyses (Fig. 3B, C; Fig. S7A). Additionally, enrichment of cell cycle-related transcription factors including E2F4 and E2F6 [56, 57], was seen in downregulated DEGs of both NCX and MGE NPCs with ‘E2F targets’ also showing significant enrichment in both regions as part of the MSigDB hallmark gene set enrichment (Figs. S7B; S8A). Furthermore, both NCX and MGE NPCs showed upregulation of apoptosis-related genes, such as positive regulation of apoptotic process (GO-BP) and apoptosis (KEGG and MSigDB hallmark) (Figs. 3B, C; S7A;S8A), in line with the increase in apoptotic cells observed after 6 h (Fig. 1L).

At 24 h post-irradiation, NCX and MGE NPCs showed enrichment of different processes, reflecting their distinct response. MGE NPCs at 24 h post-irradiation maintained an upregulation of the p53 signaling pathway and apoptosis-related processes, as revealed by both GO-BP and KEGG analyses (Fig. S6C; Fig. S7A; Table S1). Simultaneously, a downregulation in neurodevelopmental processes, including axon guidance, neuron projection guidance, and central nervous system development was observed in MGE NPCs (Fig. S6C). Furthermore, processes and pathways associated with ribosome biogenesis, spliceosome function, RNA transport, proteasome activity, and cell cycle were seen to be downregulated in NCX (Figs.S6B; S7A). E2F4, E2F6 and Myc, key regulators of cell cycle and transcriptional activity, were enriched as transcription factors among the downregulated genes in both MGE and NCX NPCs (Fig. S7B; S8A). Consistent with this, cell cycle pathways (e.g. G2-M checkpoint) were among the top pathways enriched in the downregulated NCX NPC DEGs at both 6 and 24 h, with G2-M checkpoint-related genes also showing significant enrichment in the MSigDB hallmark gene set for NCX during the same time frame (Figs. S7A; S8A). These findings corroborate our results, suggesting that NCX cells are likely undergoing prolonged cell cycle stalling while MGE cells undergo prolonged apoptosis in response to irradiation-induced damage. Importantly, we did not observe a down-regulation of E2F-dependent MCPH genes in these murine cells, as was recently found to be a human-specific response to radiation in cortical organoids [8].

Collectively, these findings from transcriptional profiling highlight both the inherent differences including prolonged cell cycle arrest in the NCX and similarities such as the role of p53 in the DNA damage response between NCX and MGE NPCs. They also reinforce our experimental observations of prolonged cell cycle arrest in NCX NPCs compared to MGE cells.

### RNA-Seq highlights cellular migration as a uniquely downregulated process in MGE versus NCX NPCs following irradiation

**Fig. 4:**
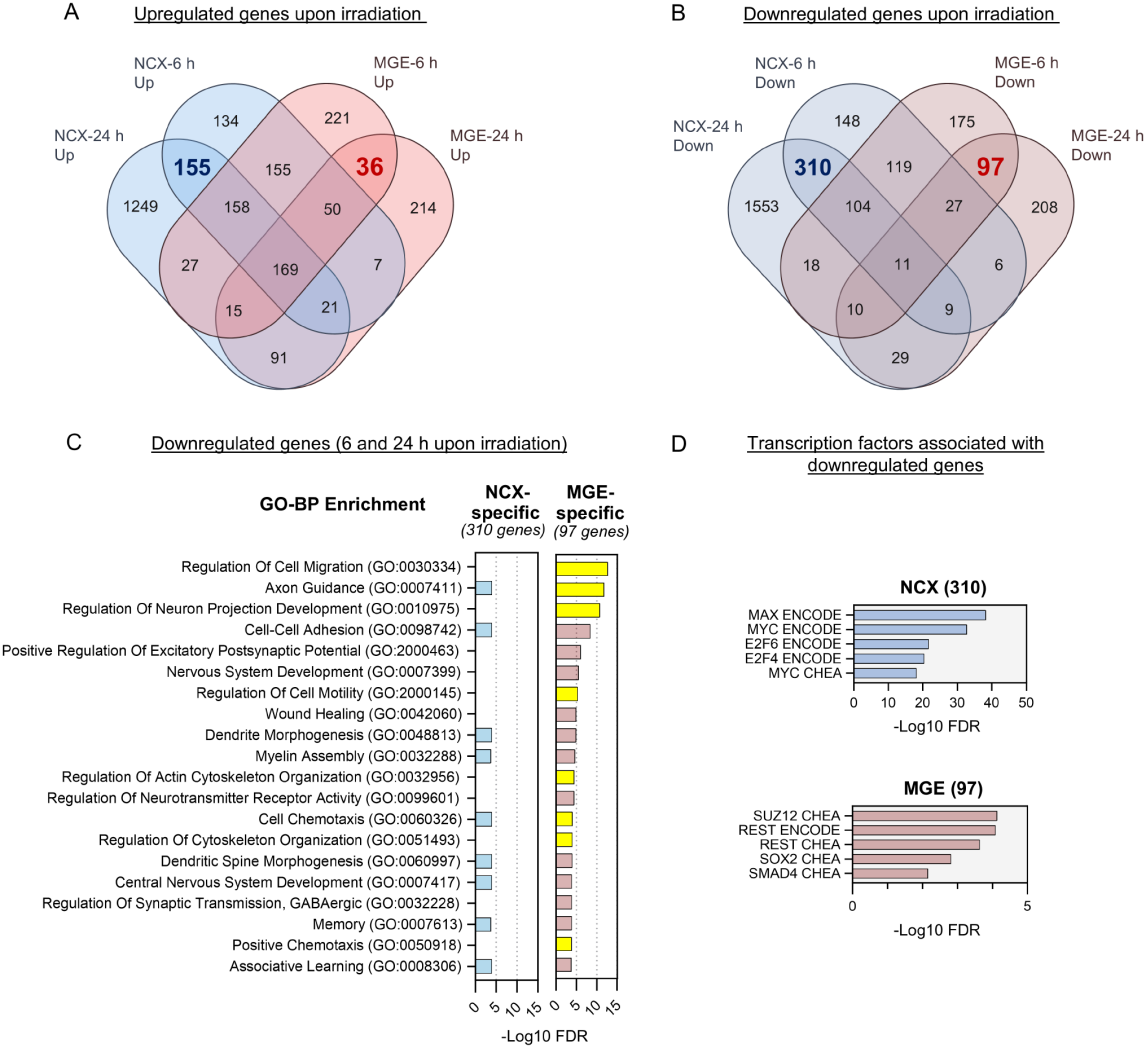
RNA-Seq highlights cellular migration as a uniquely downregulated process in MGE NPCs following irradiation. (**A**) Venn diagram showing the overlap among upregulated genes at different time points between two conditions, NCX and MGE. NCX-specific downregulated DEGs (6 and 24 h) are highlighted in blue (*n* = 155), and MGE-specific downregulated DEGs in red (*n* = 36). (**B**) Venn diagram illustrating the overlap between upregulated DEGs in NCX and MGE at both 6 and 24 h. NCX-specific downregulated DEGs (6 and 24 h) are highlighted in blue (*n* = 310), and MGE-specific downregulated DEGs in red (*n* = 97). (**C**) Comparative bar graphs showing significant enrichment of cell migration-related GO BP terms among MGE-specific downregulated genes (*n* = 97), which is not observed as enriched in NCX-specific downregulated genes (*n* = 310) following irradiation. GO BP terms related to migration process of the cells are highlighted in yellow. (**D**) Graph plots showing top transcription factors associated with NCX and MGE specific downregulated genes

To further investigate region-specific transcriptional responses, we analyzed genes that were specifically up- or downregulated in either cell type. Gene overlaps show shared and unique repression patterns between the regions over time (Fig. 4; Fig. S8B; Table S2). In NCX NPCs specifically, 155 genes were consistently upregulated, while 310 were consistently downregulated at both 6 and 24 h post-irradiation (Fig. 4A, B). In contrast, MGE NPCs specifically showed consistent downregulation of 97 genes and upregulation of 36 genes at both time points (Fig. 4A, B). While we investigated both up- and downregulated pathways (Fig. 4; Fig. S8B), migration-related processes (GO:0030334) emerged as the most enriched term among the 97 downregulated genes in MGE NPCs (Fig. 4C). Additionally, the enrichment of GO terms such as axon guidance, neuron projection development, cell-cell adhesion, actin cytoskeleton organization, and regulation of cytoskeleton organization was observed (Fig. 4C). Notably, this strong enrichment of cell migration-related terms was observed exclusively among the 97 downregulated genes in MGE NPCs and not among the 310 downregulated genes in NCX NPCs (Fig. 4C). This coincided with the enrichment of the transcription factors REST and SUZ12 among these 97 genes in MGE NPCs (Fig. 4D), which are typically associated with maintaining neural progenitor identity and neurodevelopmental processes [58–60]. In contrast, the 310 downregulated genes in NCX NPCs showed enrichment cell cycle-related transcription factors including E2F4, E2F6 and Myc (Fig. 4D). The specific downregulation of migration-related genes in MGE NPCs suggests that irradiation-induced gene repression in MGE may disrupt critical processes involved in cell migration. Interneurons, which originate in the ventral telencephalon rely on tangential and radial migration to reach their final destinations in the cortex. Interneuron migration depends on both cell intrinsic and extrinsic mechanisms involving changes in the cytoskeleton and the sensing of extracellular cues [61–63]. Thus, the observed downregulation of genes related to these pathways prompted us to further investigate the potential disruption of MGE-derived interneuron migration after irradiation.

### Prenatal irradiation delays the migration of interneurons

**Fig. 5:**
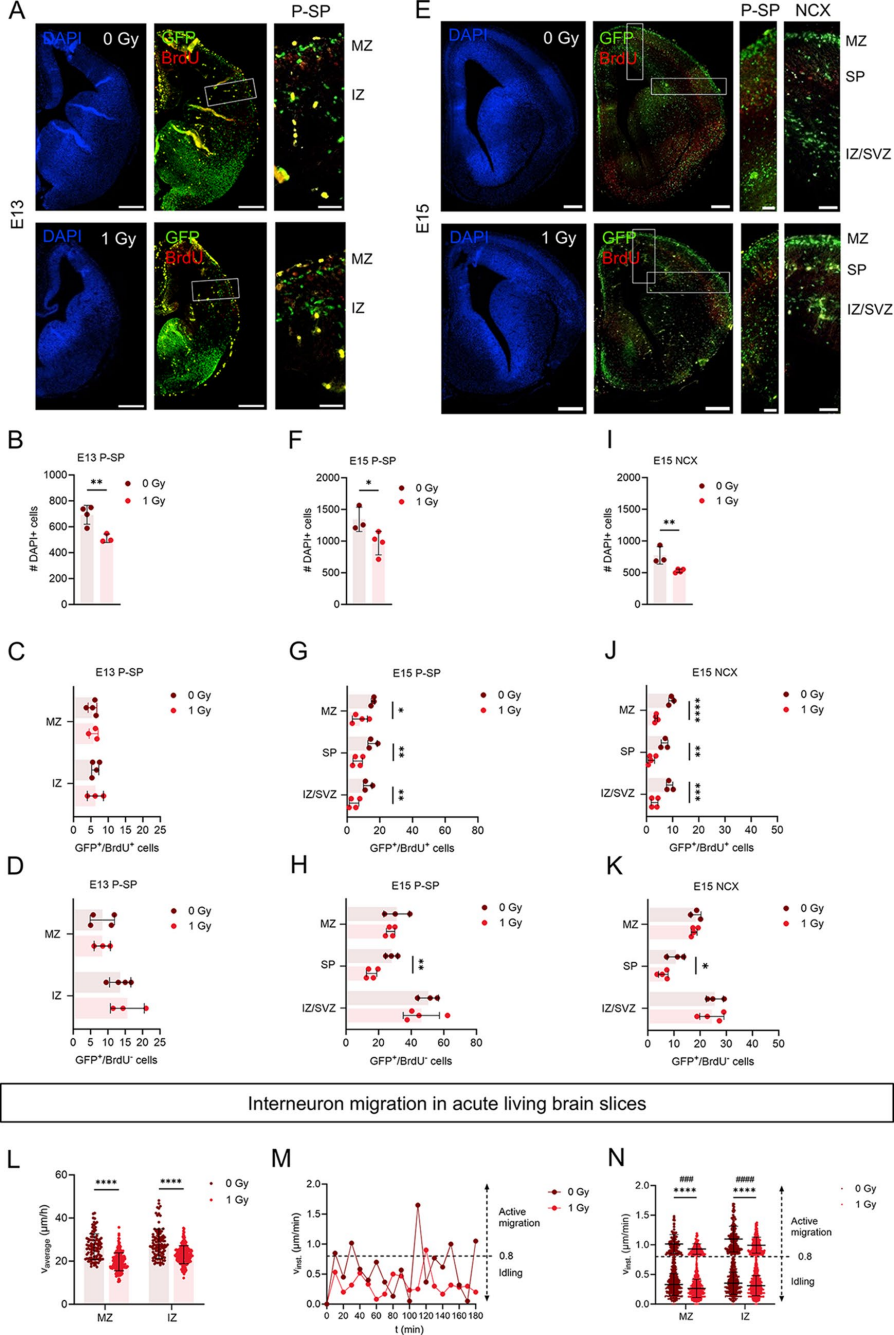
Prenatal irradiation disrupts interneuron migration. Interneuron migration was evaluated using *Nkx2.1* reporter mice (*Nkx2.1*^*eGFP/+*^) to properly distinguish eGFP-expressing interneurons in the embryonic brain. (**A**–**K**) Half an hour prior to irradiation, pregnant dams were injected with BrdU enabling to distinguish between cells that were still proliferating at the time of irradiation and their progeny (GFP^+^/BrdU^+^) and cells that were already post-mitotic at that time (GFP^+^/BrdU^−^). *n* = 3–4. Unpaired t-test or Mann-Whitney test was used. (**A**,** E**) DAPI and GFP/BrdU double staining of E13 and E15 sham (0 Gy)- and 1 Gy-irradiated brains. Scale bar = 200 μm (overview) and 50 μm (zoom-in). (**B**) Amount of DAPI-positive (DAPI+) cells in pallium-subpallium (P-SP) boundary of E13 embryos. (**C**,** D**) Amount of GFP^+^/BrdU^+^ cells and GFP^+^/BrdU^−^ corrected based on reduction in amount of DAPI + cells, in the migration streams (MZ, IZ) in the E13 P-SP. (**F**) Amount of DAPI + cells in the P-SP boundary of E15 embryos. (**G**,** H**) Amount of GFP^+^/BrdU^+^ cells and GFP^+^/BrdU^−^ corrected based on reduction in amount of DAPI + cells, in the migration streams (MZ, SP, IZ/SVZ) in the E15 P-SP boundary. (**I**) Amount of DAPI + cells in the cortex of E15 embryos. (**J**,** K**) Amount of GFP^+^/BrdU^+^ cells and GFP^+^/BrdU^−^ corrected based on reduction in amount of DAPI + cells, in the migration streams (MZ, SP, IZ/SVZ) in the E15 cortex. (**L**) Average migration speed of interneurons migrating in the MZ and IZ of E13.5 embryos. (**M**) Representative instantaneous speed in function of time of an interneuron migrating. Phases of active migration are interspersed with idling, defined as an instantaneous speed lower than a threshold of 0.8 μm/min (dotted line). (**N**) Instantaneous speed of the active (#) and idling (*) migration events of interneurons migrating in the MZ and IZ of E13.5 embryos. *N* = 100–227 cells from 3–6 embryos. Mann-Whitney test was used. IZ, intermediate zone; MZ, marginal zone; SP, subplate; SVZ, subventricular zone

To assess the potential effect of irradiation on interneuron migration we first used BrdU pulse labeling in *Nkx2.1*^*eGFP/+*^ mice, which express GFP in the lineage of cells that are born in the MGE. Half an hour prior to irradiation, pregnant dams were injected with BrdU enabling us to distinguish between cells that were still proliferating at the time of irradiation and their progeny (GFP^+^/BrdU^+^) versus cells that were already post-mitotic at that time (GFP^+^/BrdU^−^). Importantly, interneuron migration only starts after S-phase has been completed [64]. At E13, interneurons migrate in two streams, in the marginal zone (MZ) (which lies on top of the preplate (PP)) and intermediate zone (IZ), while at E15 they migrate in three streams: the MZ, subplate (SP) and IZ/subventricular zone (SVZ) [65]. Evaluation of GFP^+^/BrdU^+^ and GFP^+^/BrdU^−^ cells was performed at the pallial-subpallial (P-SP) boundary of E13 brains, when interneurons had not yet reached the dorsal cortex. At E15, both the P-SP boundary and the cortex were evaluated. A reduction in DAPI-positive cells was observed in the P-SP boundary at E13 and in both the P-SP boundary and cortex at E15 (Fig. 5A-K), reflecting the microcephalic phenotype [17, 19, 22]. While GFP^+^/BrdU^+^ or GFP^+^/BrdU^−^ cell numbers were normal at E13, irradiation led to fewer GFP^+^/BrdU^+^ cells in all migration streams and a reduction in the GFP^+^/BrdU^−^ progeny in the SP stream at E15 (Fig. 5A-K).

### Prenatal irradiation disrupts interneuron migration in acute living brain slices

To evaluate whether the apparent delayed migration was a consequence of a difference in interneuron migration speed, live cell imaging was performed on acute living brain slices. Briefly, pregnant dams were irradiated at E11 and brain slices of E13.5 *Nkx2.1*^*eGFP/+*^ embryos were imaged every 10 min for a total duration of 6 h using two-photon real time imaging (Video S1A, B). Our analysis revealed that the average migration speed was reduced in both the MZ and IZ of irradiated brain slices compared to non-irradiated controls (Fig. 5L). Interneurons typically migrate via a process called saltatory migration, which consists of cycles of nuclear pausing/idling (i.e., searching for guidance cues) and active migration (i.e., nucleokinesis) [66, 67]. To distinguish between idling and active migration, we plotted the instantaneous speed, defined as the speed between two subsequent time points. An idling threshold of 0.8 μm/min was set, corresponding to half a cell diameter (8 μm) per 10 min and confirmed by a custom-made macro developed by Gorelik and Gautreau [42]. The instantaneous speed of two representative cells demonstrates the saltatory behavior, characterized by cycles of idling (< 0.8 μm/min) and active migration (> 0.8 μm/min) (Fig. 5M). The results indicate that irradiated interneurons exhibit slower migration in both their resting and active phases (Fig. 5N). The reduction in migration speed highlights the impact of irradiation on the migratory behavior of interneurons, which may be essential for proper brain development.

### Prenatal irradiation affects the intrinsic migration machinery of interneurons

**Fig. 6:**
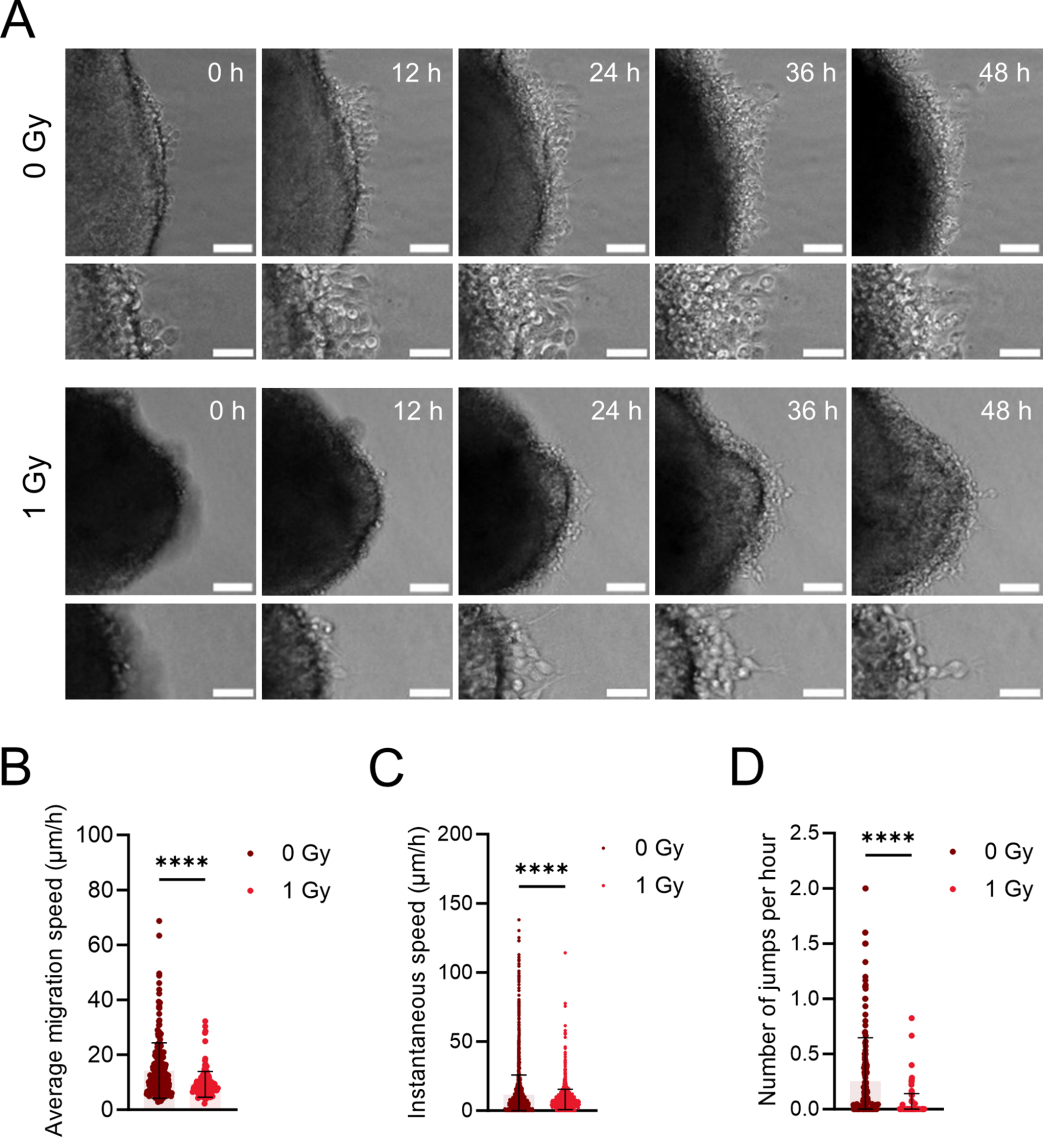
Prenatal irradiation affects the intrinsic migration machinery of interneurons. (**A**,** B**) Representative migration tracks of migrating interneurons in MGE explants. Scale bar = 10 μm (overview) and 5 μm (zoom-in). (**B**,** C**) Average and instantaneous migration speed of interneurons migrating at MGE explant edge. (**D**) Number of ‘jumps’ >15 µm per hour of interneurons migrating at MGE explant edge. N = 157–238 cells from 11–12 explants. Mann-Whitney test was used

To further investigate whether the observed effects of irradiation on interneuron migration were due to intrinsic (cell-autonomous) factors, MGE explants were used. Pregnant dams were irradiated at E11, and MGE explants were dissected at E12 and cultured in a nitrocellulose-based medium. The use of nitrocellulose minimized the presence of motogenic factors that could otherwise influence interneuron migration from the explant [68]. The migration of interneurons out of the explant was followed every 30 min for a total duration of 48 h using live imaging (Fig. 6A).

Both the average migration speed and instantaneous speed, as well as the number of jumps (i.e., active migration phases), were reduced in cells migrating out of explants from irradiated mice compared to non-irradiated controls (Fig. 6B-D). The same was observed in explants from *Trp53* cKO MGE embryos (Fig. S9), suggesting that *Trp53* is not involved in the migration deficits caused by irradiation. The disruption of migration in explants lacking extracellular cues suggested that irradiation impairs the intrinsic cellular machinery of interneurons.

### Irradiation affects regulatory components governing actin and microtubule dynamics in interneurons

**Fig. 7:**
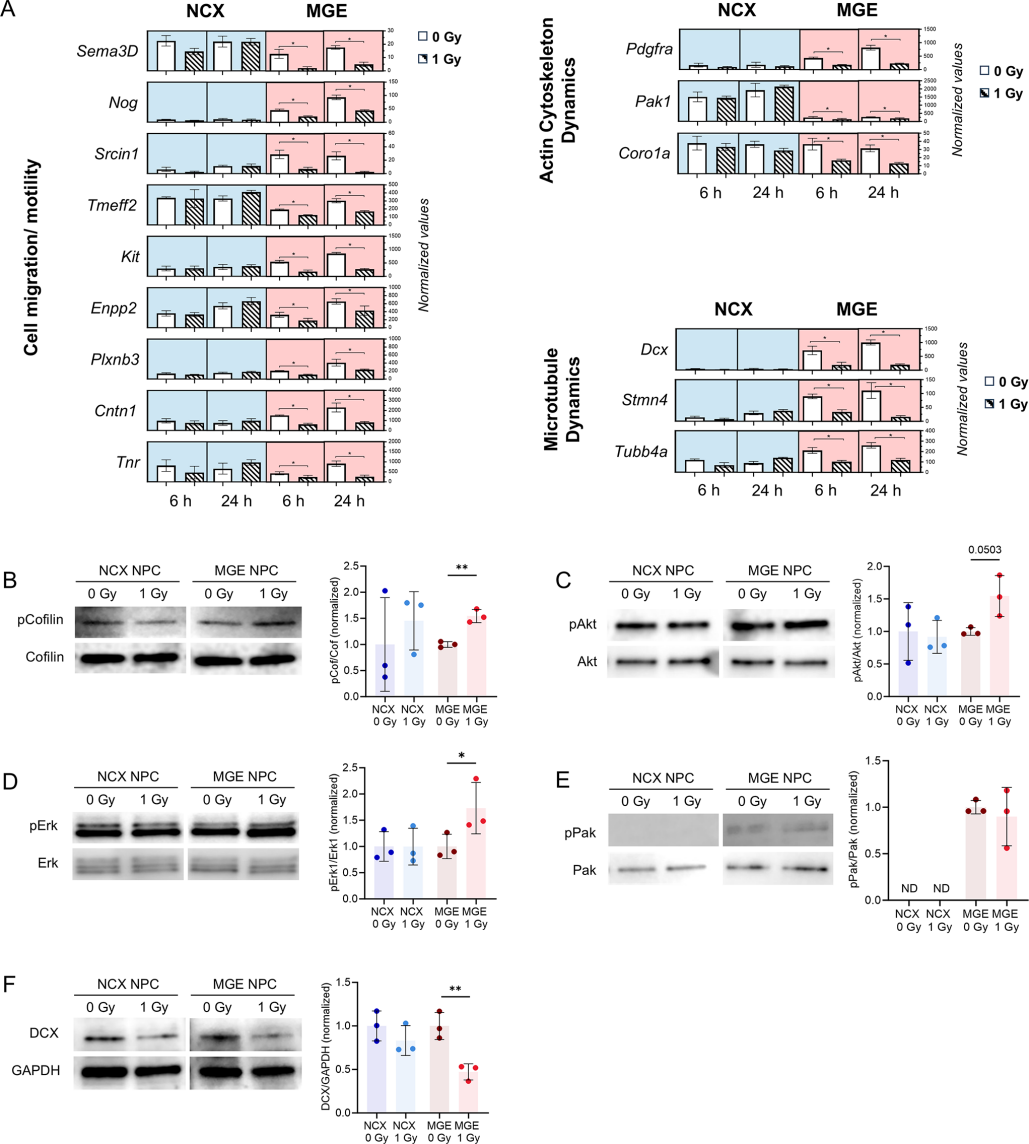
Irradiation affects regulatory components governing actin and microtubule dynamics in interneurons. (**A**) Normalized expression values of MGE-specific downregulated genes (6 and 24 h) associated with the regulation of cell migration and motility (left), as well as actin cytoskeleton (right top) and microtubule dynamics (right bottom). Statistical significance was determined using DESeq with FDR < 0.05. (**B**–**F**) Expression of proteins (pCofilin/Cofilin, pAkt/Akt, pErk/Erk, pPak/Pak, DCX) involved in interneuron migration machinery in MGE and NCX primary cell culture. *n* = 3. Paired t-test or Wilcoxon test was used

To identify potential mechanisms affecting the intrinsic cellular machinery, we revisited the RNAseq data. We found several genes involved in cell migration/motility, exhibiting notable downregulation following irradiation. Sema3D, Scrin1, Tmeff2, Enpp2, Plxnb3, Cntn1 and Tnr, showed reduced expression at both 6 and 24 h post-irradiation in MGE NPCs. Similarly, genes associated with actin cytoskeleton dynamics (Pak1 and Coro1a) and microtubule dynamics (Dcx, Stmn4, and Tubb4a) were consistently downregulated in the MGE (Fig. 7A). This widespread reduction suggests a significant disruption of underlying mechanisms related to cellular migration, including impaired cytoskeletal remodeling or microtubule stability, which are essential for the coordinated motility of interneurons [61, 62]. Given that many of the observed genes are crucial for actin cytoskeleton regulation, we performed western blot analysis on several key players in the actin signaling pathway. This analysis revealed an increased pCofilin/Cofilin ratio upon irradiation in irradiated MGE NPCs (Fig. 7B), suggesting elevated levels of inactive cofilin. Cofilin, a critical downstream regulator of actin filament turnover, is a key marker of actin dynamics, with its phosphorylation state reflecting shifts in actin polymerization. Additionally, upstream signaling showed an increased pErk/Erk and pAkt/Akt ratio in irradiated MGE NPCs, while no significant changes were observed in the pPak/Pak ratios (Fig. 7C-E). Notably, we also detected a reduction in DCX (Fig. 7F), a microtubule-associated protein with an important role in interneuron migration [69], indicating a potential disruption in microtubule dynamics that could contribute to the observed migration defects. Overall, these findings indicate that radiation caused disruptions in the regulatory components governing actin and microtubule dynamics resulting in impaired interneuron migration.

### Cortical interneuron positioning and seizure susceptibility is unaffected in prenatally irradiated young adult mice

**Fig. 8:**
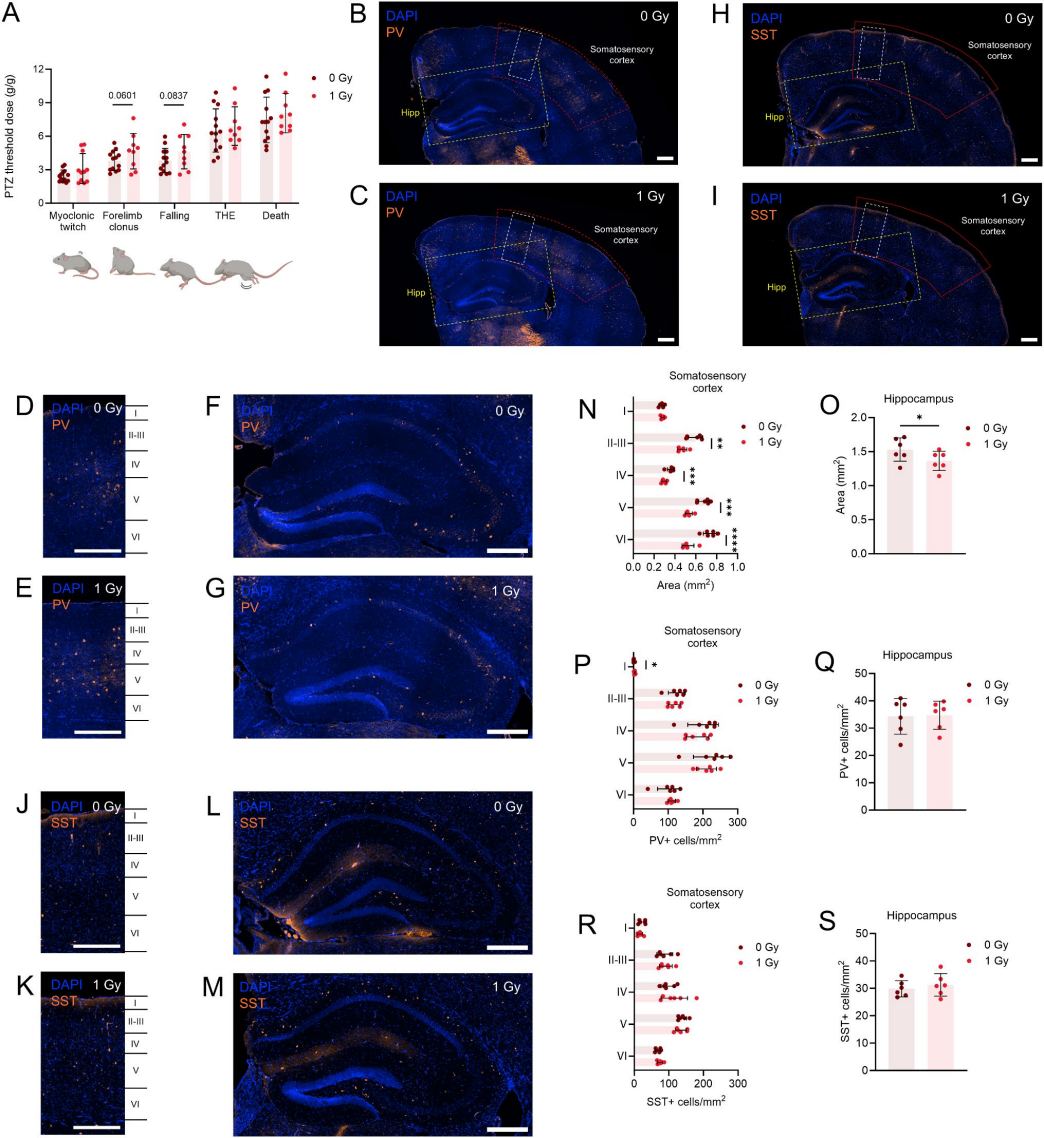
Prenatal irradiation leaves seizure susceptibility and interneuron positioning unharmed in the young adult brain. (**A**) Histogram of seizure-related behavioral alterations (Racine score) following pentylenetetrazol (PTZ) injection in P56 offspring of sham- and 1 Gy-irradiated dams. *n* = 11–13. Unpaired t-test or Mann-Whitney test was used. (**B**,** C**) Immunostaining of interneuron subtype marker parvalbumin (PV) in the P56 brain. The red area outlines the somatosensory cortex, the yellow box highlights the hippocampus. (**D–G**) Representative images of PV staining in P56 somatosensory cortex (**D**, **E**) and hippocampus (F, G). (**H**,** I**) Immunostaining of interneuron subtype marker somatostatin (SST) in the P56 brain. The red area outlines the somatosensory cortex, the yellow box highlights the hippocampus. (**J–M**) Representative images of SST staining in P56 somatosensory cortex (**J**, **K**) and hippocampus (**L**, **M**). (**N**,** O**) Area of cortical layers I, II-III, IV, V, VI in the somatosensory cortex (**N**) and hippocampus (**O**) of P56 offspring of dams which received sham- or 1 Gy-irradiation. (**P–S**) Number of PV-positive (+) (**P**, **Q**) or SST+ (**R**, **S**) interneurons in the somatosensory cortex and hippocampus of P56 offspring. Scale bar = 300 μm. *n* = 6. Unpaired t-test or Mann-Whitney test was used. Hipp, hippocampus; PV, parvalbumin; SST, somatostatin

The observed widespread apoptosis and interneuron migration defects suggested that prenatal irradiation could have long-term consequences related to an imbalance in the excitatory and inhibitory activity, such as epilepsy, which may result from an excitation-inhibition imbalance in the brain. Previous studies showed that prenatal irradiation in rats and humans, as well as interneuron migration defects in mice and humans, can increase the risk of (epileptic) seizures [70–73]. To test the hypothesis that irradiation at an early stage of neurogenesis in mice might increase seizure susceptibility, we conducted an acute seizure test using the chemoconvulsant pentylenetetrazol (PTZ) which induces generalized seizures. PTZ was administered via the tail vein to both irradiated and non-irradiated offspring at post-natal day 56 (P56) to assess seizure thresholds for the following behaviors: myoclonic ear twitch, forelimb clonus, falling, tonic hind limb extension (THE) and death (Fig. 8A). No differences in PTZ seizure thresholds were observed between irradiated and non-irradiated mice (Fig. 8A). This suggested that, contrary to our expectations, prenatal irradiation did not affect the seizure susceptibility and, therefore, does not affect the establishment of the cortical circuitry during brain maturation. Thus, we assessed interneuron numbers and distribution in P56 mice that were irradiated prenatally. In agreement with the microcephalic phenotype, most cortical layers as well as the hippocampus were reduced in size (Figs. S1E; 8B-O). However, the positioning of parvalbumin and somatostatin-positive cells, the two main types of cortical interneuron subtypes, were unaffected, except for a small increase in the number of parvalbumin-positive cells in cortical layer I (Fig. 8D-G, J-M, P-S). Overall, this showed that despite excessive apoptosis at early time points following irradiation, and a reduced speed of tangential migration, ultimately interneurons are able to populate dorsal areas in sufficient numbers such that excitation/inhibition balance seems unaffected.

## Discussion

Exposure to maternal insults like irradiation or ZIKV infection, showing significant overlap in phenotypic and transcriptional changes [74], induces DNA damage in the developing brain and has been associated with neurodevelopmental disorders including microcephaly [71, 75]. While most studies primarily focused on the DNA damage response and perturbations in developing excitatory (NCX) neurons [2, 19], the impact on inhibitory (MGE) neurons remains largely unexplored. In this study, prenatal irradiation was used to induce DNA damage in both NCX and MGE NPCs. We found that both cell types activate a p53-dependent DNA damage response, although NCX cells were shown to undergo prolonged cell cycle arrest while MGE cells exhibit sustained apoptosis. In addition, our findings show that irradiation also disrupts interneuron migration, a key developmental process. This defect appears to result from radiation-induced alterations in the intrinsic migration machinery, particularly in the regulation of the actin and microtubule cytoskeleton.

### The differential DNA damage response in the NCX and MGE

Several studies have demonstrated that various progenitors in the developing brain are affected differently by prenatal disturbances. For example, dorsal and ventral OPCs are differentially affected by prenatal DNA damage. In a Citron-kinase (CITK) knockout model of microlissencephaly, where CITK, an MCPH gene associated with cytokinesis defects, DSB accumulation and p53-dependent apoptosis when deficient [76, 77], dorsal OPCs undergo apoptosis, while ventral OPCs exhibit senescence [15]. Similar vulnerabilities were seen in WT OPCs treated with cisplatin, a cross-linking agent that induced DNA damage [15]. Irradiation at E14.5 and LPS-induced maternal inflammation also highlight regional brain sensitivities, with differing apoptotic responses between the dorsal telencephalon and LGE, and greater hypoxia susceptibility in the NCX compared to the LGE or MGE [78, 79]. *Magoh*-deficient mice, which develop microcephaly due to extensive DNA damage and cell death, show prolonged mitosis and disrupted neurogenesis in both dorsal and ventral progenitors [80–83]. Our immunostainings and enrichment analysis revealed both distinct and overlapping DNA damage responses in MGE and NCX cells. Both regions showed DSB repair, cell cycle arrest, and apoptosis, with the NCX experiencing a prolonged arrest and MGE exhibiting prolonged apoptosis (Fig. 1; Figs. S7A; S8A), underscoring differences in their respective response.

One possible explanation for this is the different cell cycle dynamics between dorsal and ventral progenitors. Cell cycle data shows that while the total cell cycle length is longer in the GE compared to the NCX at E11-12 [34, 35], it begins to shorten from E13 onward [84, 85]. The earlier shift of terminal neurogenic divisions from the VZ to the SVZ in the GE, starting at E11.5 [35], facilitates the rapid expansion necessary for producing a large number of migrating interneurons. In contrast, the NCX experiences a longer phase of progenitor proliferation, with the shift in terminal divisions starting at E14 [35], allowing for more regulated cortical neuron production [85]. Shorter G1 phases in early neural progenitors increase their susceptibility to genotoxic stress [16]. The distinct cell cycle characteristics of the NCX and MGE likely influence their responses to DNA damage, however, future research is needed to further explore these differences, as data on MGE cell cycle length is limited.

Our results indicate that approximately 48% of cells in the NCX and 20% in the MGE underwent apoptosis at 6 h post-irradiation (Fig. 1F). Although these percentages suggest higher apoptosis in the NCX compared to the MGE, it is important to consider that these regions differ in their composition and contain varying proportions of proliferative zone cells [35, 86]. At E11.5, the NCX primarily consists of the VZ and the cortical plate [87]. In contrast, the MGE is comprised of the VZ, SVZ, and the mantle zone, where postmitotic cells start migrating to their final locations [88]. Since the NCX lacks both the SVZ and mantle zone at E11.5, deriving conclusions from a direct comparison of apoptotic cell numbers between the MGE and NCX is not appropriate. However, 24 h after irradiation the number of apoptotic cells dramatically reduced in the NCX in comparison to 6 h. In the MGE, the level of apoptosis remained high (Figs. 1F and 2L, Fig. S6C;S8A). This sustained apoptosis is accompanied by earlier resolution of cell cycle arrest at 6 h in the MGE, suggesting that MGE cells may attempt to resume the cell cycle more quickly, potentially before fully repairing the damage. In contrast, cells in the NCX exhibit a slower exit from cell cycle arrest which may reflect a more cautious or thorough DNA repair process in the NCX, allowing cells to avoid prolonged apoptosis once damage is resolved. Alternatively, it is possible that NCX cells are still in the process of repairing damage at 24 h and, as a result, have not yet entered apoptosis. Overall, these findings suggest that different brain regions respond to DNA damage with distinct temporal dynamics, potentially influenced by region-specific factors such as cell type, function, or developmental stage. Additionally, the selection of DNA repair pathways in both the MGE and NCX, which is closely linked to the cell cycle and regulated by region- and cell type-specific expression of DNA repair machinery [89, 90], may further contribute to these observed region-specific differences.

The DNA damage response in primary NCX and MGE cell cultures aligned to a certain extent with our observations in the embryonic brain. However, several differences between in vivo and in vitro conditions were noted. For instance, cell cycle arrest was more pronounced in vivo, and apoptosis remained elevated at 24 h in irradiated versus non-irradiated conditions in vivo, but not in vitro (Fig. 1). Additionally, apoptosis was also less pronounced in primary cultures compared to the embryonic brain (Fig. 1). One possible explanation is that cell cycle dynamics differ significantly between in vivo and in vitro conditions. Cells in culture can continue proliferating for extended periods, whereas in vivo, environmental signals promote differentiation and halt proliferation. Finally, the dynamics of the DNA damage response, particularly the p53 response, also differ. In vivo, p53 target genes are highly induced at 2 h post-irradiation, with reduced activation at 6 h and beyond. In contrast, in vitro conditions show minimal activation at 2 h, peaking at 6 h post-irradiation [19, 51].

The tumor suppressor p53 plays a critical role in mediating the DNA damage response, particularly in radiation-induced cell cycle arrest and apoptosis [14, 19, 47, 51]. Our findings in cKO mice lacking *Trp53* in the NCX or MGE confirm that p53 is essential for these processes (Fig. 2). The reduced cell cycle arrest and apoptosis in *Trp53* cKO mice highlights p53’s central role in managing cellular responses to radiation-induced damage [7, 14, 47]. Consistent with this, both NCX and MGE NPCs showed p53-dependent genes being excessively enriched at 6 h post-irradiation, along with activation of the PI3K-Akt and MAPK signaling pathways (Fig. S7A, B; Fig. S8), which are known to interact with p53 and are crucial for promoting cell survival, proliferation, and stress responses following radiation-induced damage [91, 92]. This underscores a role of p53 in preventing genomic instability.

### Impaired interneuron migration without lasting alterations in the young adult brain

The developmental trajectory of interneurons, from stem cell to mature interneuron, includes tightly regulated processes such as differentiation, migration, synaptogenesis and cortical connection [93]. Disruptions at any stage, whether due to intrinsic or extrinsic factors, can lead to interneuron dysfunction. Both human and animal studies have shown that mutations in disease risk genes [94–97] or adverse prenatal environment [29–32] can cause abnormalities in interneuron development, contributing to neurodevelopmental disorders. Ectopic cell populations and a migration delay have been observed in the cortex of prenatally irradiated humans, rats and mice [98–101].

Our transcriptomic analysis did not reveal changes in migration-related genes in irradiated NCX cells (Figs. 4C and 8A). Prenatal irradiation in rats did not affect cytoskeletal elements, however, it did lead to a loss of N-CAM, a cell adhesion molecule, and delayed neuronal migration within the cortical plate [100]. Our earlier work found ectopic neurons in the NCX following E11 irradiation, attributed to premature neuronal differentiation rather than migration defects [19]. While further investigation into premature neuronal differentiation is needed, our current results support radiation-induced premature neuronal differentiation, indicated by the upregulation of pathways related to nervous system development, synapse organization, axonogenesis and synaptic transmission (Fig. S6B) which are linked to neuronal differentiation.

Some of the GFP^+^/BrdU^−^ interneurons that reached the P-SP boundary at E13 may have begun their migration shortly after irradiation at E11.5 [102]. Thus, at the time of irradiation at E11 these cells have exited the cell cycle [64] and therefore became less likely to undergo DNA damage-induced apoptosis, as is reflected by the absence of apoptotic cells in the mantle zone of the MGE (Figs. 1E and 2K). However, also their migration machinery could have been affected which would explain the observed reduction in migration speed in brain slices at E13.5. By E15, a significant reduction in GFP^+^/BrdU^+^ cells in all three migration streams (MZ, SP and IZ/SVZ) suggests that radiation-induced DNA damage, cell cycle arrest and apoptosis in the MGE likely disrupted the proliferation and migration of these cells. The decrease in GFP^+^/BrdU^−^ cells at E15 could reflect either interneurons that initiated migration shortly after irradiation, or progeny of irradiated cells that lost BrdU through multiple rounds of division. These cells might still be impacted by irradiation, either due to intrinsic defects in their migration machinery or because changes in the irradiated brain environment such as alterations in the extracellular matrix other environmental factors essential for proper cell migration. Additionally, the observed migration defects, including alterations in actin and microtubule regulatory components, may contribute to the delay in interneuron migration observed in E15 brains. This suggests that both intrinsic and extrinsic factors may play a role in the observed migration abnormalities following irradiation.

Our findings demonstrate significant migration impairments in both acute brain slices and MGE explants (Figs. 5, 6 and 7), which is supported by the downregulation of migration-associated genes in irradiated MGE cells (Figs. 4C and 7A). This suggests that intrinsic cellular mechanisms, particularly those related to cytoskeletal dynamics, drive these deficits. Irradiated MGE NPCs showed elevated inactive cofilin (pCofilin) levels possibly due to enhanced Erk activation (Fig. 7), via LIMK, stabilizing actin filaments and impairing cell migration [103]. Concomitantly, an increase in pAkt activation was also observed (Fig. 7), linked to alterations in PI3/Akt signaling, which affects cytoskeletal remodeling [104, 105]. While Pak1 typically phosphorylates LIMK to regulate cofilin [106], we observed no significant changes in Pak1 phosphorylation, despite reduced RNA expression (Fig. 7). Our findings align with previous studies on migration defects in hypoxic human ventral forebrain organoids and Arl13b mutant interneurons, where altered pAkt, pErk, and cAMP levels impacted cell migration [107, 108]. Elevated cAMP can hinder interneuron branching, while decreased Erk phosphorylation delays migration [69, 109]. Although Erk activation generally promotes migration [109], it also catalyzes cofilin phosphorylation, disrupting F-actin dynamics, as seen in heart tissue [110]. In Down syndrome GABAergic neurons, increased Pak1 levels contributed to migration defects through elevated pCofilin [111]. In our irradiated MGE cells, however, despite reduced Pak1 expression, phosphorylated Pak1 levels remained unchanged, suggesting a complex interaction between Pak1 and Erk signaling. In summary, we hypothesize that increased pErk activity, along with reduced Pak1 levels, elevates inactive cofilin via LIMK, disrupting actin dynamics and impairing interneuron migration after irradiation.

Beyond the regulation of actin cytoskeleton components, our results also show a reduction in DCX, suggesting a potential disruption in microtubule dynamics. In agreement with our findings, DCX knockout has been linked to slowed interneuron migration and branching defects [112, 113]. While previous studies report varied migration dynamics, they both support the notion that reduced DCX expression leads to abnormal interneuron migration, aligning with our findings.

Our results suggest that the radiation-induced migration impairments and cytoskeletal alterations are influenced by the activity of transcription factors REST and SUZ12 which where enriched among the MGE-specific downregulated genes (Fig. 4D; Fig. S7B). Both REST and SUZ12 are key neurodevelopmental regulators and play (in)direct roles in neuronal migration [60, 114]. REST, a transcriptional repressor, controls the expression of numerous genes involved in neuronal differentiation and cytoskeletal organization It recruits the PRC2 complex, including SUZ12 and EZH2, to silence target genes in non-neuronal cells and neural progenitors [59, 115]. REST has been shown to both inhibit and promote migration depending on the context [114, 116, 117]. PRC2 is also known as a regulator of neuronal migration through gene expression control [118], and SUZ12 knockdown has been linked to increased cell migration via Erk1/2 pathway activation [119]. In contrast, interneuron migration was not affected in MGE explants from irradiated p53 cKO mice, indicating that the migration defect is independent of p53 signaling in response to DNA damage. Further research is needed to clarify the specific pathways through which these transcription factors modulate interneuron migration. However, we hypothesize that the downregulation of REST and SUZ12 targets following irradiation may contribute to the observed migration defects by directly or indirectly altering the expression of genes involved in regulating interneuron migration.

Despite migration defects, no differences in interneuron positioning were observed in irradiated offspring at P56 (Fig. 8), highlighting the potential plasticity of brain development in certain contexts. Similarly, a previous study showed that ectopic BrdU-labeled cells, present in the cortex 3 days after E14 irradiation, were absent by 8 weeks [98], suggesting the activation of compensatory mechanisms that may promote cellular clearance and restoration of normal cortical architecture. In contrast, E16 irradiation altered BrdU-labeled cell distribution at 6 weeks [120]. Contrary to our expectations of increased seizure susceptibility based on epidemiological studies [3, 121], we found no differences post-irradiation (Fig. 8). Although previous animal studies on seizure susceptibility following prenatal irradiation have reported conflicting results, with some showing increased seizure responses and others showing decreased responses after prenatal irradiation exposure [70, 121–123], our outcome opens up exciting avenues for future research into how protective mechanisms might mitigate such risks. Additionally, earlier studies from our lab showed that E11 irradiation caused mild cognitive defects and brain size reduction [20, 124]. One of the most obvious findings from these studies was an impaired learning and memory observed in Morris water maze experiments. Animals that were irradiated with 1 Gy at E11 showed decreased escape latency that was associated with a reduction in the use of spatial search strategies [124]. Learning and memory are highly dependent on a correct E/I balance in the prefrontal cortex and hippocampus [125, 126]. It is therefore interesting to note that long-term potentiation in the hippocampus was affected in mice irradiated at E11 [127]. In this study, we found no differences in the distribution of PV + and SST + interneurons in the hippocampus of irradiated mice, but many other reasons may explain changes in LTP including differences in synaptic strength or neuromodulation.

Although irradiation leads to DNA damage and migration defects, compensatory mechanisms, such as plasticity in other cell types or network-wide synaptic adaptations, may reveal how the brain maintains stability in neural circuits despite developmental challenges. Also, the massive acute apoptosis that occurs in the first day(s) after irradiation, may be followed by a reduced number of cells that die during subsequent, physiological waves of programmed cell death, which is a normal process during embryonic and early postnatal brain development [28, 128]. While no postnatal alterations were detected, further research is needed to explore those compensatory mechanisms and other potential neurodevelopmental outcomes, such as changes in electrophysiological properties or functional synapses caused by radiation-induced disturbances. Exploring these compensatory mechanisms could further uncover new therapeutic targets that enhance brain plasticity.

## Conclusion

In conclusion, our study provides comprehensive insights into the region-specific DNA damage responses and migration defects induced by prenatal irradiation in the developing mouse brain. Both NCX and MGE cells exhibited the classical p53-dependent DNA damage response, however, the prolonged cell cycle arrest in NCX cells and sustained apoptosis in the MGE indicate a differential response towards irradiation. In contrast, interneuron migration was disrupted due to intrinsic alterations, specifically involving the regulation of actin and microtubule components. The observed impairments in interneurons highlight the complex effects of DNA damage-inducing factors during neurodevelopment. Future research is needed to provide a more comprehensive understanding of the mechanisms underlying radiation-induced migration defects and to determine the long-term effects of prenatal irradiation on brain electrophysiology and behavior, particularly in the context of neurodevelopmental disorders. These findings enhance our understanding of how early developmental insults impact brain development and contribute to neurodevelopmental disorders. They also highlight the importance of investigating molecular pathways that could potentially mitigate these effects.

## Limitations of the study

We acknowledge that this study has certain limitations and leaves several questions unanswered. First, the models we used to study interneuron migration allow for a detailed examination of nucleokinesis, which is a crucial component of interneuron movement, however, this analysis was not performed here. Furthermore, the absence of a feeder layer in the explants may have restricted interneuron migration out of the explants, making it more challenging to measure their migration speed. Moreover, cell-extrinsic mechanisms, which along with the cell-intrinsic machinery plays a crucial role in interneuron migration, may also be altered by irradiation. However, their potential influence on interneuron migration was not investigated. Additionally, our study did not address the radial migration of progenitors of excitatory neurons in the NCX, an important aspect of brain development that warrants further investigation. Finally, it is uncertain to what extent our results from mice can be extrapolated to human brain development, given the potential differences in species-specific mechanisms.

## Electronic supplementary material

Below is the link to the electronic supplementary material.

Supplementary Material 1

Supplementary Material 2

Supplementary Material 3

Supplementary Material 4

Supplementary Material 5

Supplementary Material 6

## Data Availability

RNA-seq data generated in this study are available at ArrayExpress (accession number E-MTAB-14528). The datasets on DE genes in MGE and NCX NPCs are available as Supplementary Tables. Any additional information required to reanalyze the data reported in this paper is available from the lead contact upon reasonable request.
