# Correction: Interneuron migration impairment and brain region-specific DNA damage response following irradiation during early neurogenesis in mice

**DOI:** 10.1007/s00018-025-05749-y

**Published:** 2025-06-19

**Authors:** Lisa Berden, Nicholas Rajan, André Claude Mbouombouo Mfossa, Isabeau De Bie, Emre Etlioglu, Mohammed Abderrafi Benotmane, Mieke Verslegers, Najat Aourz, Ilse Smolders, Jean-Michel Rigo, Bert Brône, Roel Quintens

**Affiliations:** 1 Radiobiology Unit, Nuclear Medical Applications Institute, Belgian Nuclear Research Centre (SCK CEN), Mol, Belgium; 2 Laboratory for Neurophysiology, BIOMED Research Institute, UHasselt, Hasselt, Belgium; 3 4BRAIN, Department of Head and Skin, Faculty of Medicine and Health Sciences, Ghent University, Ghent, Belgium; 4 Preclinical Sciences and Translational Safety, Johnson & Johnson IM, Beerse, Belgium; 5 Research Group Experimental Pharmacology (EFAR), Center for Neurosciences (C4N), Faculteit Geneeskunde en Farmacie, Vrije Universiteit Brussel (VUB), Brussels, Belgium

**Correction: Cellular and Molecular Life Sciences (2025) 82:118**

10.1007/s00018-025-05643-7

In this article the wrong Supplementary file was originally published with this article; it has now been replaced with the correct file.

The original article has been corrected.

## Electronic supplementary material

Below is the link to the electronic supplementary material.

Supplementary Material 1 (DOCX 40.4 MB)

