# Supplementary material for: Interneuron migration impairment and brain region-specific DNA damage response following irradiation during early neurogenesis in mice
Source: Cell Mol Life Sci. 2025 Mar 17;82(1):118. doi: 10.1007/s00018-025-05643-7 (PMC11914712; doi:10.1007/s00018-025-05643-7)
Supplement: Supplementary file 1 — Supplementary Material 1 [file 18_2025_5643_MOESM1_ESM.docx]

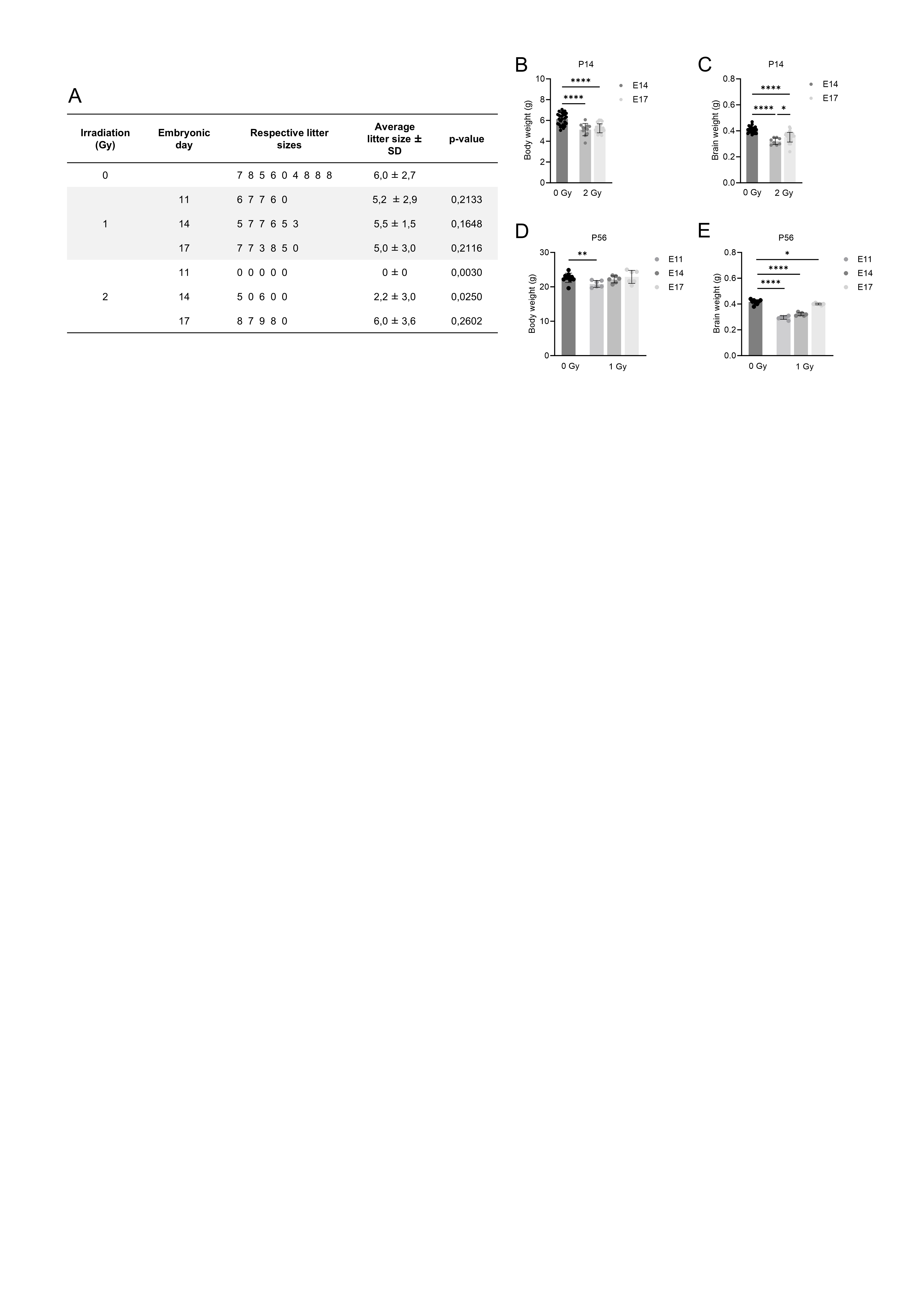


**Figure S1. E11 is most sensitive to prenatal irradiation. (A)** Litter size of pregnant dams sham-irradiated (0 Gy), irradiated with 1 Gy or 2 Gy at embryonic day (E) 11, 14 or 17. n = 5-9. Mann-Whitney test was used to compare 0 Gy with each experimental group. **(B, C)** Body and brain weight of P14 offspring (male and female) of sham- and 2 Gy-irradiated dams. n = 11-32. One-way ANOVA test followed by Tukey’s test for multiple comparisons was used. **(D, E)** Body and brain weight of P56 offspring (male and female) of sham- and 1 Gy-irradiated dams. n = 6-10. One-way ANOVA test followed by Tukey’s test for multiple comparisons was used.


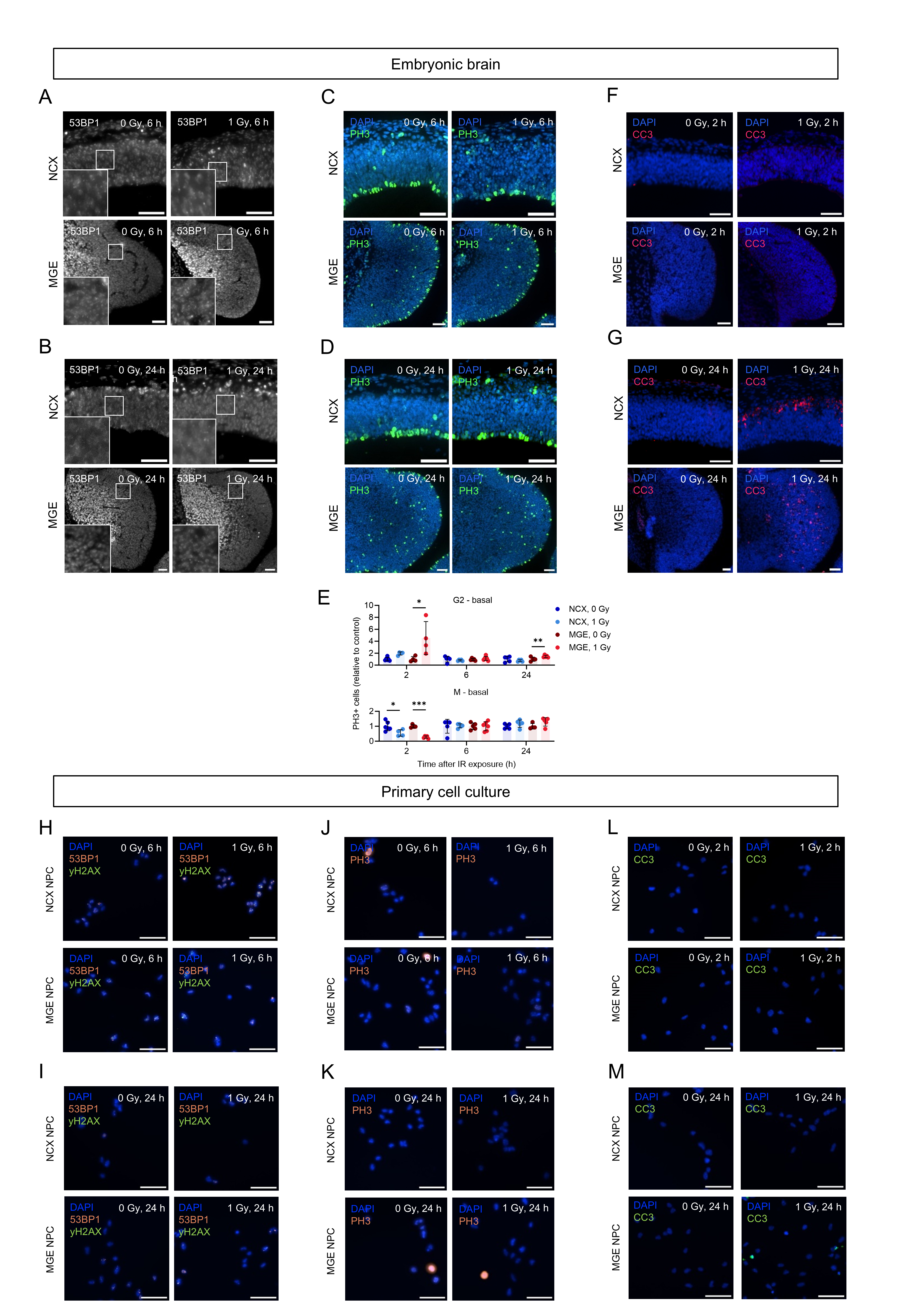


**Figure S2. Representative images of DDR markers at additional time points and basal cell cycle arrest in the NCX and MGE. (A-D, F-G)** Immunostaining of DSB marker 53BP1 (A, B), late G2/M phase marker PH3 (C, D) and apoptosis marker CC3 (F, G) in NCX and MGE, 6 and 24 h (A-D) or 2 and 24 h (F, G) post-irradiation. **(E)** PH3-positive cells (G2/M) relative to control at 2, 6 and 24 h post-irradiation in the basal zone of NCX and MGE. n = 4-6. One-way ANOVA test followed by Tukey’s test for multiple comparisons or Kruskal-Wallis with Dunn’s test for multiple comparisons was used. **(H-M)** Immunostaining of DSB marker 53BP1 (H, I), late G2/M phase marker PH3 (J, K) and apoptosis marker CC3 (L, M) in NCX and MGE primary cell cultures, 6 and 24 h (H-K) or 2 and 24 h (J, M) post-irradiation. Scale bar = 50 µm.


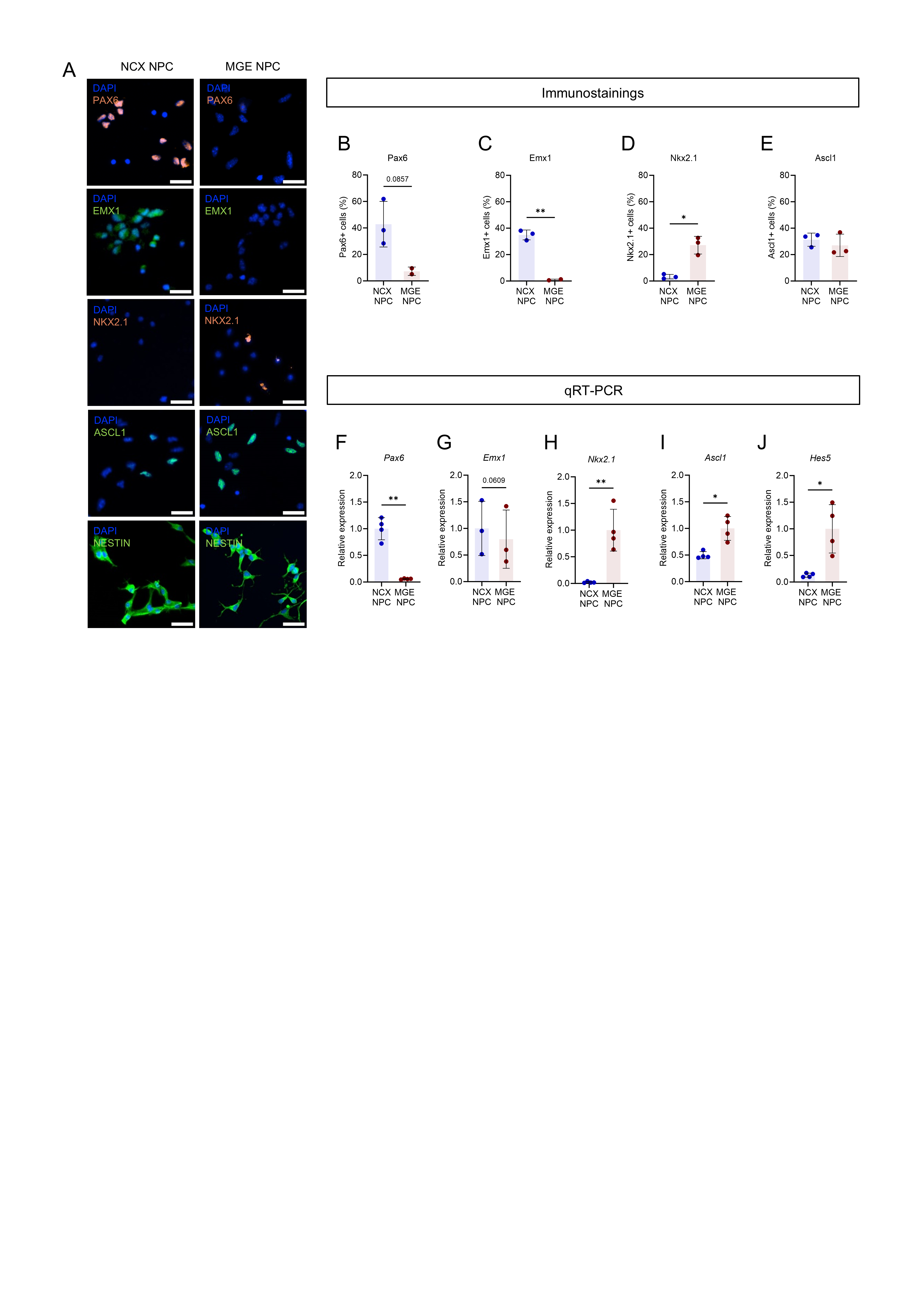


**Figure S3. Validation of NCX and MGE lineage of primary cell cultures. (A)** Immunostainings of dorsal and ventral markers, and neural stem/progenitor cell marker Nestin in NCX and MGE NPCs. Scale bar = 40 µm. **(B-E)** Percentage of Pax6, Emx1, Nkx2.1 and Ascl1-positive NCX and MGE NPCs validated by immunostainings. NCX NPCs: n/N_Pax6_ = 3/3034; n/N_Emx1_ = 3/3091; n/N_Nkx2.1_ = 3/8522; n/N_Ascl1_ = 3/4634 and MGE NPCs: n/N_Pax6_ = 2/1296; n/N_Emx1_ = 2/1154; n/N_Nkx2.1_ = 3/2833; n/N_Ascl1_ = 3/1149. **(F-J)** Relative expression of D/V markers in NCX and MGE NPCs validated by qRT-PCR. n = 3-4. Paired t-test was used.


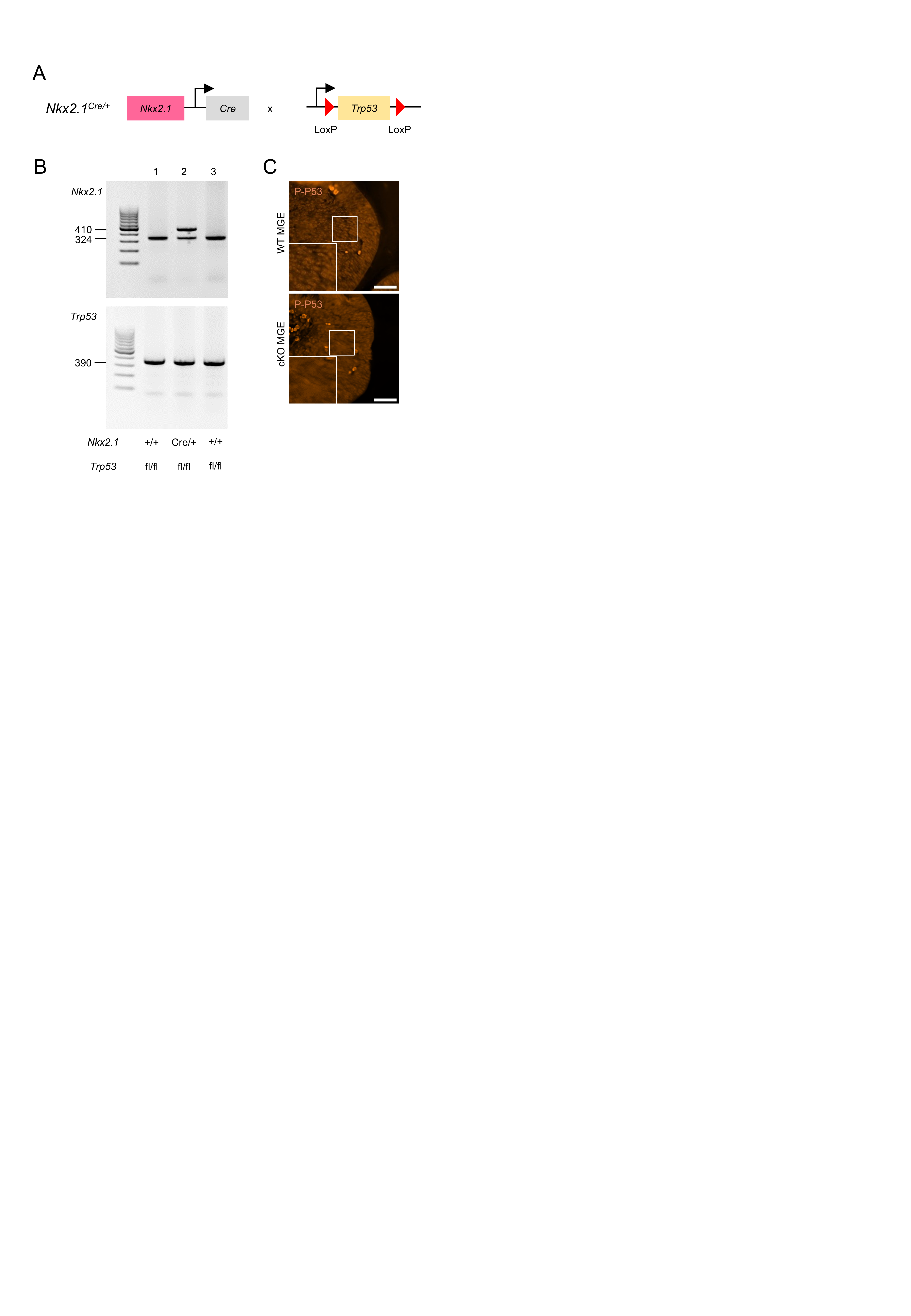


**Figure S4. Validation of cKO MGE.** **(A)** Strategy for the generation of Trp53 conditional knockout animals in the MGE using Nkx2.1-Cre. **(B)** Representative PCR results of Nkx2.1^Cre/+^; Trp53^fl/fl^ (cKO MGE) and Nkx2.1^+/+^; Trp53^fl/fl^ (WT MGE) mice. The 324- and 410-bp bands indicate the wild-type and mutant alleles, respectively. **(C)** Phosphorylated p53 (p-p53) staining in Trp53 WT and cKO MGE 2 h post-irradiation (1 Gy). Scale bar = 50 µm.


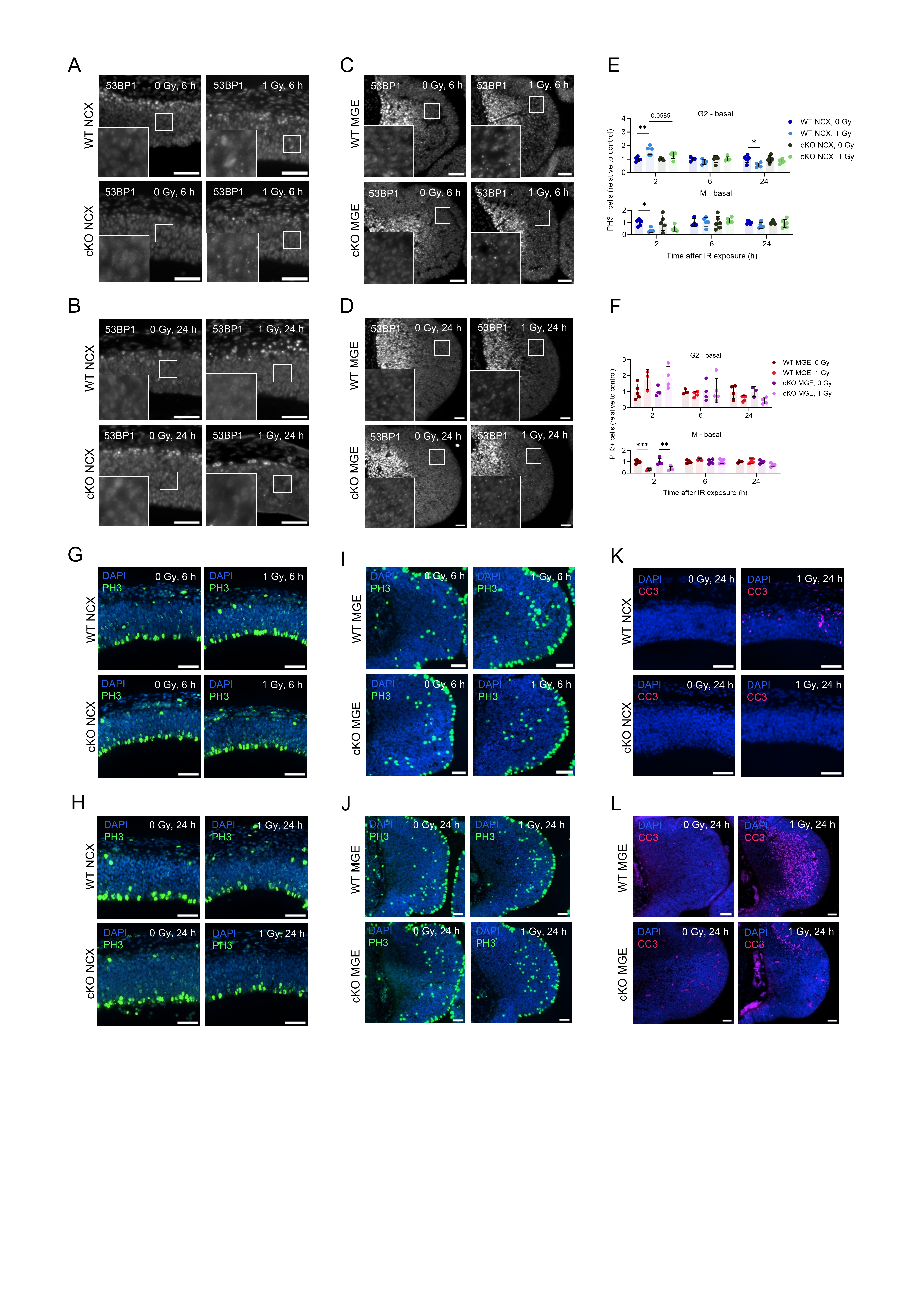


**Figure S5. Representative images of DDR markers at additional time points and basal cell cycle arrest in Trp53 WT and cKO embryos.** **(A-D)** Immunostaining of DSB marker 53BP1. Representative images of NCX and MGE of WT, cKO NCX and cKO MGE, 6 and 24 h post-irradiation. **(E, F)** Relative amount of PH3-positive cells in G2 and M-phase (relative to control) in the basal zone of NCX of WT and p53 cKO NCX embryonic brains (E) and MGE of WT and p53 cKO MGE embryonic brains (F). n = 3-6. One-way ANOVA test followed by Tukey’s test for multiple comparisons or Kruskal-Wallis with Dunn’s test for multiple comparisons was used. **(G-J)** Immunostaining of late G2/M phase marker PH3, 6 and 24 h post-irradiation. **(K, L)** Immunostaining of apoptosis marker CC3, 24 h post-irradiation. Scale bar = 50 µm.


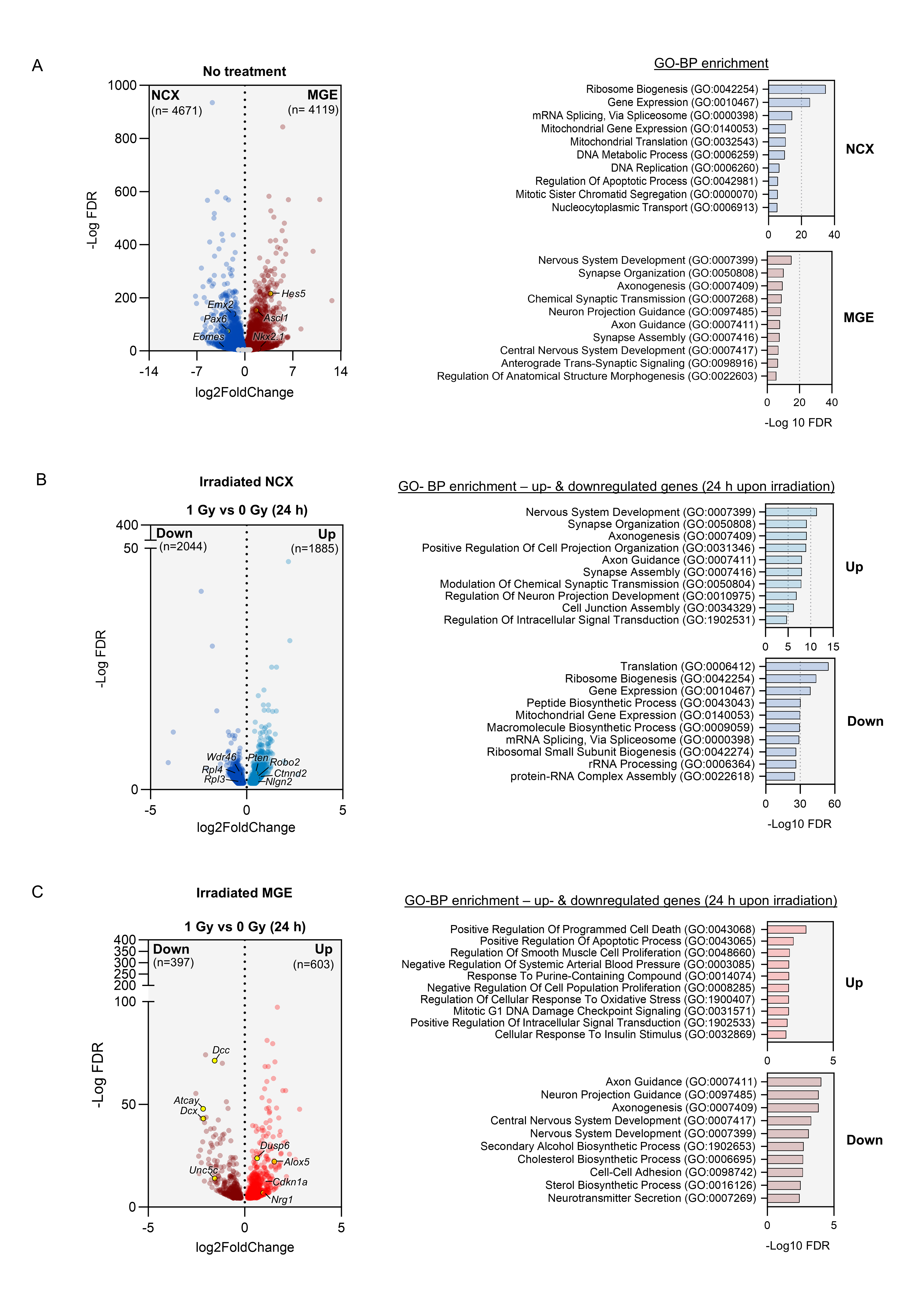


**Figure S6. GO-BP enrichment reveals the distinct identity and p53-dependent DNA damage response of NCX and MGE NPCs, 24 h post-irradiation. (A) (Left)** Volcano plot showing DEGs (FDR < 0.05) between NCX and MGE NPCs in the non-irradiated condition. DEGs were identified in both NCX (n = 4,671) and MGE (n = 4,119) NPCs under untreated conditions. **(Right)** GO BP enriched terms associated with NCX NPC DEGs (top) and MGE NPC DEGs (bottom). Relevant DEGs are highlighted in the volcano plot on the left. **(B) (Left)** Volcano plot showing significantly upregulated (n = 1,885, light blue) and downregulated (n = 2,044, dark blue) DEGs (FDR < 0.05) between 1 Gy irradiated and sham (0 Gy) NCX NPCs at 24 h. **(Right)** Enrichment analysis of GO BP terms associated with upregulated and downregulated DEGs identified in NCX NPCs at 24 h. Relevant DEGs are highlighted in the volcano plot on the left. **(C) (Left)** Volcano plot of significant DEGs (FDR < 0.05) between 1 Gy irradiated and sham (0 Gy) MGE NPCs at 24 h post-irradiation. Upregulated DEGs are shown in light red (n = 397), while downregulated DEGs are shown in dark red (n = 603). **(Right)** Enrichment analysis of GO BP terms for upregulated and downregulated DEGs identified in MGE NPCs at 24 h, with relevant DEGs highlighted in the volcano plot on the left.


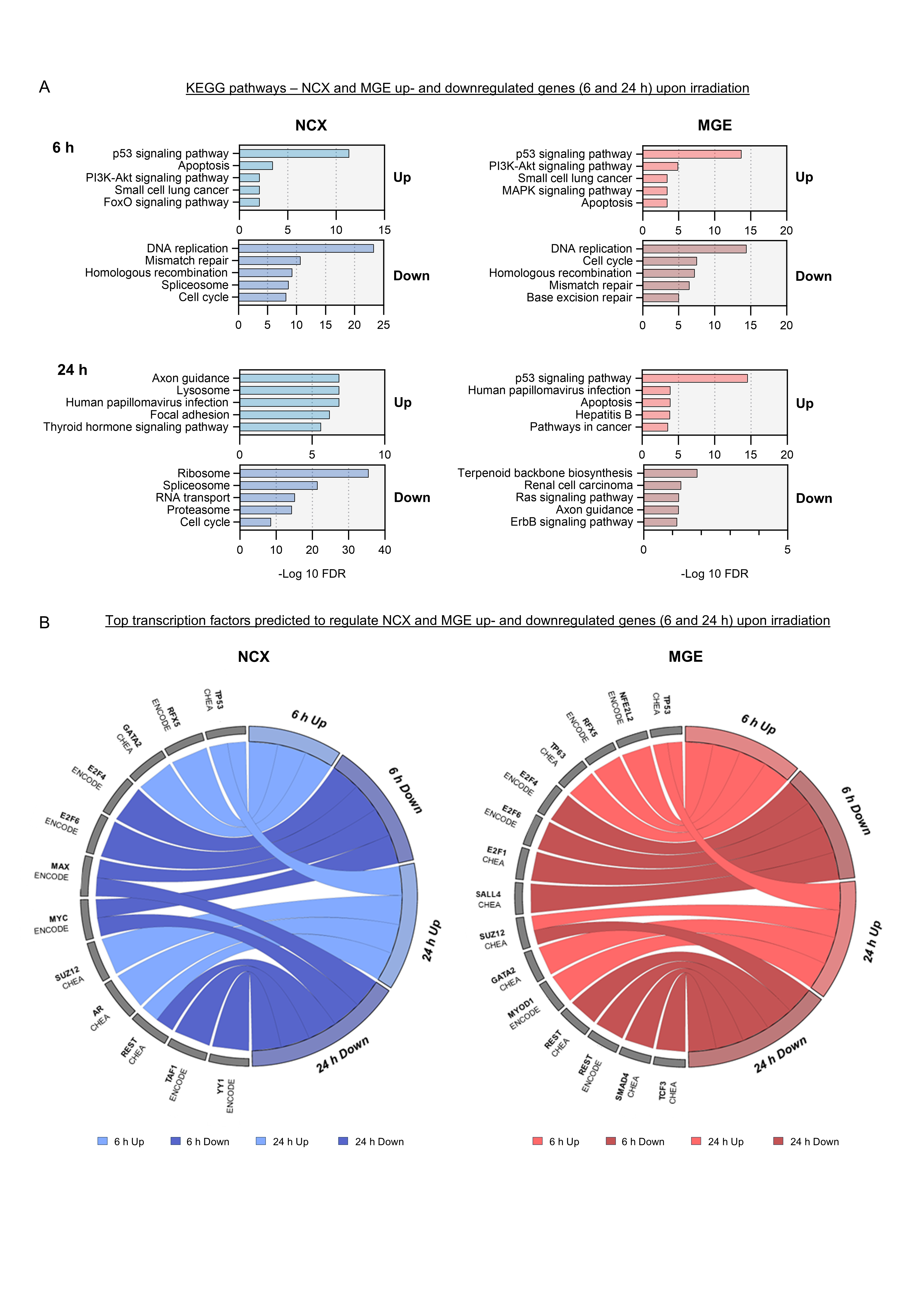


**Figure S7. Transcriptional profiling reveals divergent cell cycle arrest patterns and transcription factors regulating enriched genes in NCX and MGE NPCs upon irradiation. (A)** Comparative analysis of KEGG pathways associated with upregulated and downregulated genes in NCX and MGE NPCs at 6 and 24 h post-irradiation. The panels display the top enriched KEGG pathways for both upregulated and downregulated genes in NCX (left) and MGE (right) derived NPCs following irradiation. The data are separated into 6 h (top) and 24 h (bottom) post-irradiation time points. **(B)** Enrichment analysis of predicted transcriptional regulators for upregulated and downregulated DEGs in NCX and MGE NPCs post-irradiation. Enrichment data was obtained from the ChIP-X Enrichment Analysis (ChEA) and Encyclopedia of DNA Elements (ENCODE) databases.


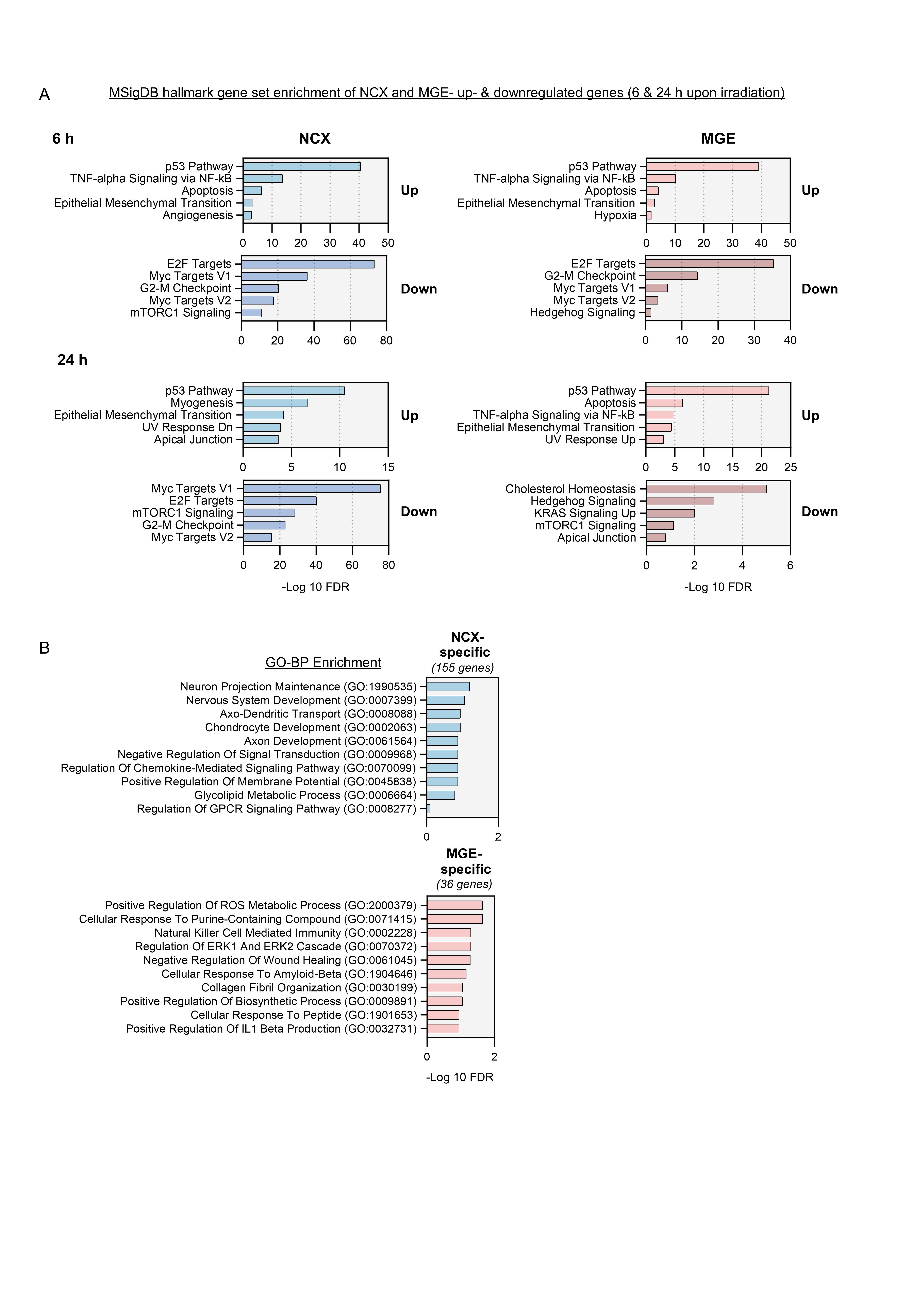


**Figure S8. Comparative MSigDB hallmark gene set enrichment and GO analysis of up- and/or downregulated genes in NCX and MGE NPCs post-irradiation. (A)** Comparative analysis of MSigDB hallmark gene set enrichment associated with upregulated and downregulated genes in NCX and MGE NPCs at 6 and 24 h post-irradiation. The panels display the top enriched MSigDB hallmark genes for both upregulated and downregulated conditions in NCX (left) and MGE (right) derived NPCs following irradiation. The data are separated into 6 h (top) and 24 h (bottom) post-irradiation time points. **(B)** GO enrichment bar plots represent the top GO biological processes enriched for the upregulated genes specifically in NCX (top) and MGE (bottom).


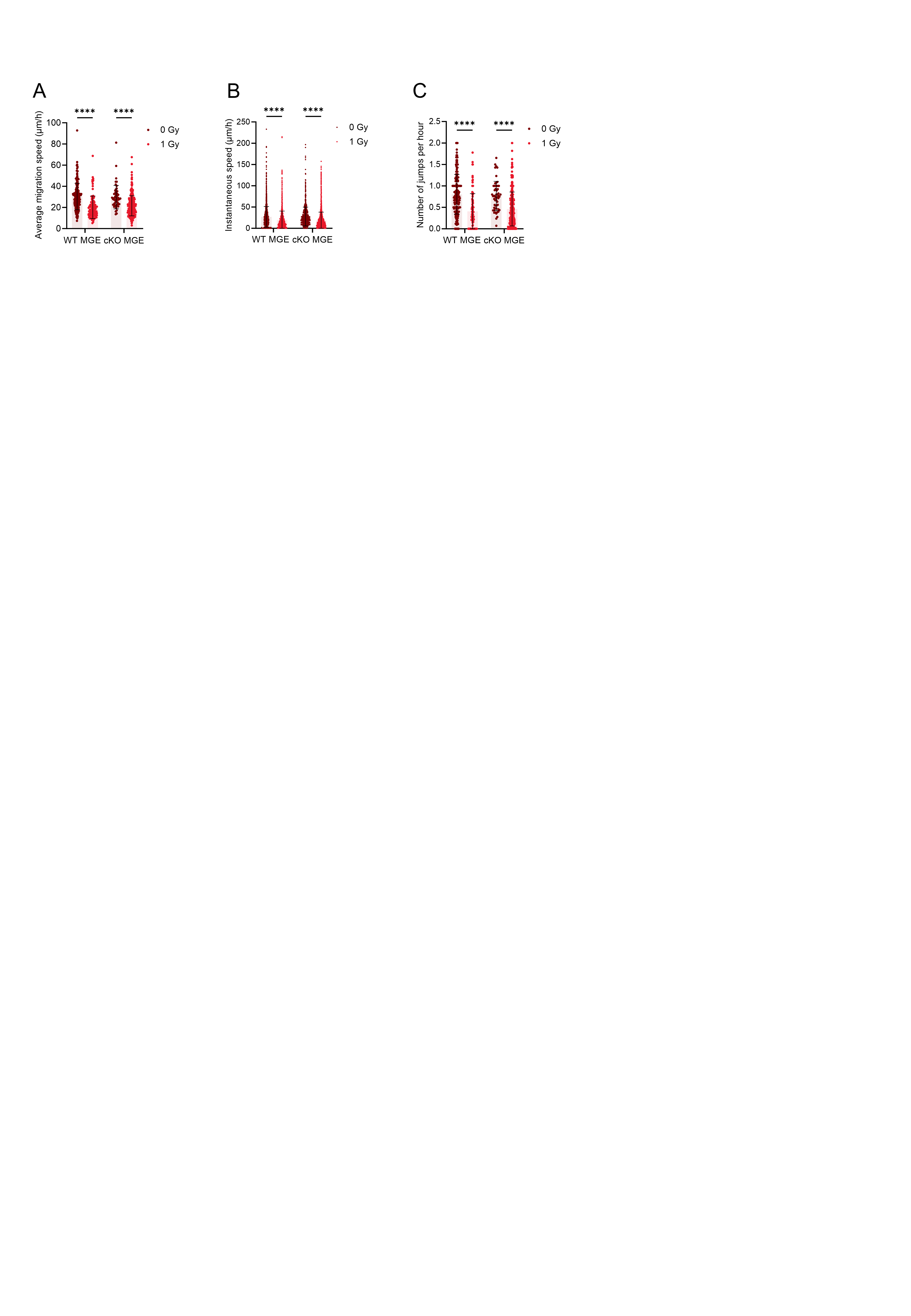


**Figure S9. Trp53 is not involved in the interneuron migration discrepancies observed upon irradiation. (A, B)** Average and instantaneous migration speed of interneurons migrating at MGE explant edge, isolated from WT and p53 cKO MGE embryonic brains. **(D)** Number of ‘jumps’ >15 µm per hour of interneurons migrating at MGE explant edge, isolated from WT and p53 cKO MGE embryonic brains. N = 55-328 cells from 3-15 explants. Kruskal-Wallis test was used.

**Video S1. Interneuron migration in acute living brain slices, related to Figure 5 M-N. (A, B)** Movies of migrating interneurons in E13.5 acute living brain slices of non-irradiated (A) and irradiated (B) embryo. Scale bar = 100 µm.
